# *Discobola* Osten Sacken, 1865 (Diptera, Limoniidae) in China: Taxonomic Review, Updated Distribution, and DNA Barcoding

**DOI:** 10.3390/insects16080845

**Published:** 2025-08-15

**Authors:** Shuo Ma, Liying Dai, Hanhuiying Lv, Yuqing Wei, Xiao Zhang

**Affiliations:** Shandong Engineering Research Center for Environment-Friendly Agricultural Pest Management, College of Plant Health and Medicine, Qingdao Agricultural University, Qingdao 266109, China; mashuo3241@163.com (S.M.); dailiying29@163.com (L.D.); hanlv_xm@163.com (H.L.); wyq05218@163.com (Y.W.)

**Keywords:** taxonomy, Chinese fauna, revision, new record, identification key, DNA barcodes, *COI*

## Abstract

The genus *Discobola* Osten Sacken, 1865 from China is taxonomically reviewed using an integrative approach that combines detailed morphological examination and molecular analysis. *Discobola parvispinula* (Alexander, 1947), a species widely distributed across the Palaearctic region, is newly recorded from China. Updated distributional data are presented for species known from China: *D. annulata* (Linnaeus, 1758), *D. armorica* (Alexander, 1942), *D. margarita* Alexander, 1924, and *D. taivanella* (Alexander, 1930). Detailed redescriptions and illustrations, including intraspecific morphological variation, are provided for these species. An identification key to Chinese *Discobola* species is also presented. Geographical analysis reveals a higher species richness in southern China and the Qinghai–Tibet region, with a progressive decline toward northern and northwestern China. The first DNA barcode reference library for Chinese *Discobola* is established, comprising 15 mt *COI* sequences from five species. These sequences, analyzed alongside an additional 101 mt *COI* sequences from *Discobola* species in other countries, show that intraspecific divergence within the genus remains below 7.4%, while interspecific divergence ranges from 7.6% to 17.7%. These findings provide important insights into the taxonomy, species delimitation, and biogeography of *Discobola* in China, contributing to a more comprehensive understanding of *Discobola* diversity across the region.

## Simple Summary

The crane fly genus *Discobola* Osten Sacken, 1865, a small and morphologically distinctive taxon within the family Limoniidae, is characterized by its conspicuously patterned wings—often marked with extensive maculae, spots, or ocelli—as well as notable body coloration. Species delimitation within this genus remains challenging due to the considerable variability in external coloration and the inadequate or ambiguous descriptions of male hypopygial structures in many original taxonomic treatments. Currently, *Discobola* is known to occur in only three provincial-level regions of China, with five previously recorded species. Based on over two decades of extensive fieldwork across multiple provinces of China, numerous *Discobola* specimens have been collected, leading to significant updates in the Chinese fauna of this genus, including the documentation of newly recorded species and expanded distributional data for previously known species. This study integrates a comprehensive taxonomic revision with DNA barcoding analysis to improve the accuracy of species identification in Chinese *Discobola*. By combining extensive sampling, morphological examination, and molecular data, this research contributes to a deeper understanding of the diversity, intraspecific morphological variation, biogeographical patterns, and molecular identification of *Discobola* in China.

## 1. Introduction

The family Limoniidae is one of the most species-rich groups within Diptera, comprising over 11,000 recognized taxa [1], and represents a lineage highly diverse in terms of taxonomy and ecology. Within this family, *Discobola* Osten Sacken, 1865 constitutes a relatively small and specialized genus. Members of *Discobola* are often characterized by distinctive body coloration, particularly the wings, which are typically extensively maculate, spotted, or ocellate. Body coloration of insects is known to serve a variety of adaptive functions, including predator avoidance, aposematism, thermoregulation, and mate selection [2]. The wing pigmentation observed in *Discobola* species is presumed to be adaptive, though the specific ecological and evolutionary significance remains to be investigated in detail. Morphologically, adult *Discobola* can be distinguished by several diagnostic features, including a distinctly elongated and narrow pronotum, tarsal claws bearing a prominent subbasal tooth accompanied by two or three smaller additional teeth, and the presence of a supernumerary crossvein near the apex of vein A_1_ [3]. The male hypopygium also exhibits distinct morphological specialization, bearing two pairs of gonostyli: the inner gonostylus is broadly oval or rounded and possesses a well-developed rostral prolongation, while the outer gonostylus is slender, heavily sclerotized, and typically hook-shaped [4,5]. Larvae and pupae of *Discobola* are primarily saproxylic, developing in decaying wood and fungal substrates [5,6,7,8,9,10].

The first species of the genus *Discobola* was described by C. Linnaeus in 1758 as *Tipula annulata* [11]. Between 1854 and 1972, a total of 26 valid *Discobola* crane flies were described globally [1], with 17 of these attributed to the prolific work of C.P. Alexander [4,12,13,14,15,16,17,18,19,20,21,22,23,24,25]. After 1972, only a single species was added to the genus over the next five decades—a new taxon described from New Zealand in 2006 [26]. Currently, the genus *Discobola* comprises 28 recognized taxa (species and subspecies) worldwide, of which 13 are from the Australasian/Oceanian region, 10 from the Palaearctic, 7 from the Oriental, 3 from the Nearctic, and 1 from the Neotropic region [1]. Five *Discobola* species have been recorded in China, occurring in only three provincial-level regions: *D. acurostris* (Alexander, 1943) from Sichuan, *D. annulata* (Linnaeus, 1758) and *D. margarita* Alexander, 1924 from Taiwan, *D. armorica* (Alexander, 1942) from Xizang, and *D. taivanella* (Alexander, 1930) from both Sichuan and Taiwan [1]. Despite the broad distribution of the genus across the Northern Hemisphere, the *Discobola* fauna of China has remained poorly understood due to a longstanding lack of systematic taxonomic investigation. This gap in knowledge has significantly hindered the accurate assessment of species diversity and biogeographical patterns within the region.

The identification of *Discobola* species has long been hindered by their complex and variable body coloration, as well as the lack of detailed morphological descriptions of the male hypopygium in many original taxonomic accounts. These limitations have posed significant challenges for accurate species delimitation within the genus, highlighting the need for more comprehensive and integrative taxonomic studies. DNA barcoding, based on a fragment of the mitochondrial (mt) cytochrome c oxidase subunit I (*COI*) gene, offers a rapid and reliable method for species identification. It is unaffected by morphological polymorphism, sexual dimorphism, or life stage [27,28,29,30] and has proven effective in resolving species boundaries across numerous dipteran groups [31,32,33,34,35,36,37,38,39,40,41,42,43,44,45,46,47,48,49,50,51,52]. This technique has also been applied successfully to various crane fly lineages [29,53,54,55,56,57,58], offering a powerful complement to traditional morphological taxonomy and evolutionary systematics. However, to date, only three identified *Discobola* species from Australia, Canada, Finland, Germany, Norway, and United States have been represented in public DNA sequence databases [59], and no comprehensive DNA barcode library exists for the Chinese fauna of this genus. This underscores the urgency of developing a reference database for *Discobola* species to support future taxonomic and biodiversity research.

To enhance the understanding of *Discobola* crane flies in China and address the current gap in DNA barcode coverage for this genus, we initiated a comprehensive project with the objectives of collecting *Discobola* specimens across China, identifying species based on morphological characteristics, and establishing a DNA barcode reference library for the Chinese fauna. Specimens from multiple localities were examined, resulting in the identification of five *Discobola* species. Among them, *D. annulata* (Linnaeus, 1758), *D. armorica* (Alexander, 1942), *D. margarita* Alexander, 1924, and *D. taivanella* (Alexander, 1930) represent known species for which distributional data in China have been significantly updated. In addition, *D. parvispinula* (Alexander, 1947), previously reported from Eastern Europe, Russia, Kazakhstan, and Japan [1], was recorded from China for the first time. This study provides a comprehensive review of the *Discobola* species in China, including the newly recorded taxon. Furthermore, the first DNA barcode reference library for Chinese *Discobola* is established, comprising 15 mitochondrial *COI* sequences representing five species.

## 2. Materials and Methods

### 2.1. Specimen Collection, Observation, and Description

Between 2002 and 2024, 79 specimens for this study were collected by different entomologists from 17 provincial-level regions of China (Beijing, Fujian, Gansu, Guangxi, Guizhou, Hebei, Henan, Hunan, Inner Mongolia, Liaoning, Shaanxi, Shanxi, Sichuan, Taiwan, Xizang, Yunnan, and Zhejiang) and deposited in the Entomological Museum of Qingdao Agricultural University, Shandong, China (QAU). Type specimens of *Discobola* deposited in the National Museum of Natural History, Smithsonian Institution, Washington, DC, USA (USNM), were also examined. Genitalic preparations were prepared by macerating the apical portion of the abdomen in cold 10% sodium hydroxide (NaOH) solution for 12–15 h to facilitate examination of internal structures. Observations and illustrations were conducted using a ZEISS Stemi 2000-C stereomicroscope (Oberkochen, Germany), and photographs were taken with Canon EOS 5D and 90D digital cameras equipped with macro lenses. Specimens were soaked in 75% ethanol during morphological examination to enhance visualization of coloration and structural details. The morphological terminology mainly followed Cumming and Wood (2017) [60], while the terminology for venation followed de Jong (2017) [61]. The following abbreviations in figures were used: aed = aedeagus; cerc = cercus; goncx = gonocoxite; hyp vlv = hypogynial valve; i gonst = inner gonostylus; o gonst = outer gonostylus; pm = paramere; rp = rostral prolongation; st = sternite; tg = tergite.

### 2.2. DNA Extraction, Amplification, and Sequencing

In this study, DNA sequences were obtained from 15 *Discobola* specimens (Table 1). All specimens for DNA extraction were preserved in absolute ethanol and stored at −20 °C for long-term preservation at Qingdao Agricultural University. Genomic DNA was extracted from thoracic muscle tissue using the TIANamp Genomic DNA Kit (TIANGEN, Beijing, China), following the manufacturer’s protocol. The DNA barcode region, corresponding to the mt *COI* gene, was amplified via PCR using the universal primers LCO1490 (5′-GGGTCAACAAATCATAAAGATATTGG-3′) and HCO2198 (5′-TAAACTTCAGGGTGACCAAAAAATCA-3′) [62]. Each PCR reaction was conducted in a 25.0 μL reaction volume containing 12.5 μL of Taq PCR Master Mix, 1.0 μL of genomic DNA, 1.0 μL of each primer (LCO1490 and HCO2198), and 9.5 μL of ddH_2_O. The thermal cycling protocol consisted of an initial denaturation at 94 °C for 4 min; followed by 30 cycles of 94 °C for 30 s, 45 °C for 30 s, and 72 °C for 1 min; and a final extension at 72 °C for 10 min. Successful PCR products were purified and subsequently sequenced by Sangon Biotech (Shanghai, China).

### 2.3. Molecular Analysis

A total of 118 mt *COI* sequences were included in the phylogenetic analysis (Table S1), comprising 15 newly generated sequences from five Chinese species and 103 sequences retrieved from the Barcode of Life Data System (BOLD) database [59]. Codon-based sequence alignment was conducted using ClustalW implemented in MEGA 12 [63]. Phylogenetic reconstruction was performed using the Maximum Likelihood (ML) method under the Kimura 2-parameter (K2P) model in MEGA 12. The robustness of the inferred tree topology was assessed through 1000 bootstrap replicates. Genetic distances among the *Discobola* sequences were calculated using the K2P model in MEGA 7 [64], providing estimates of both intra- and interspecific divergences.

## 3. Results and Discussion

### 3.1. Taxonomic Review of Chinese Discobola

**Figure 1:**
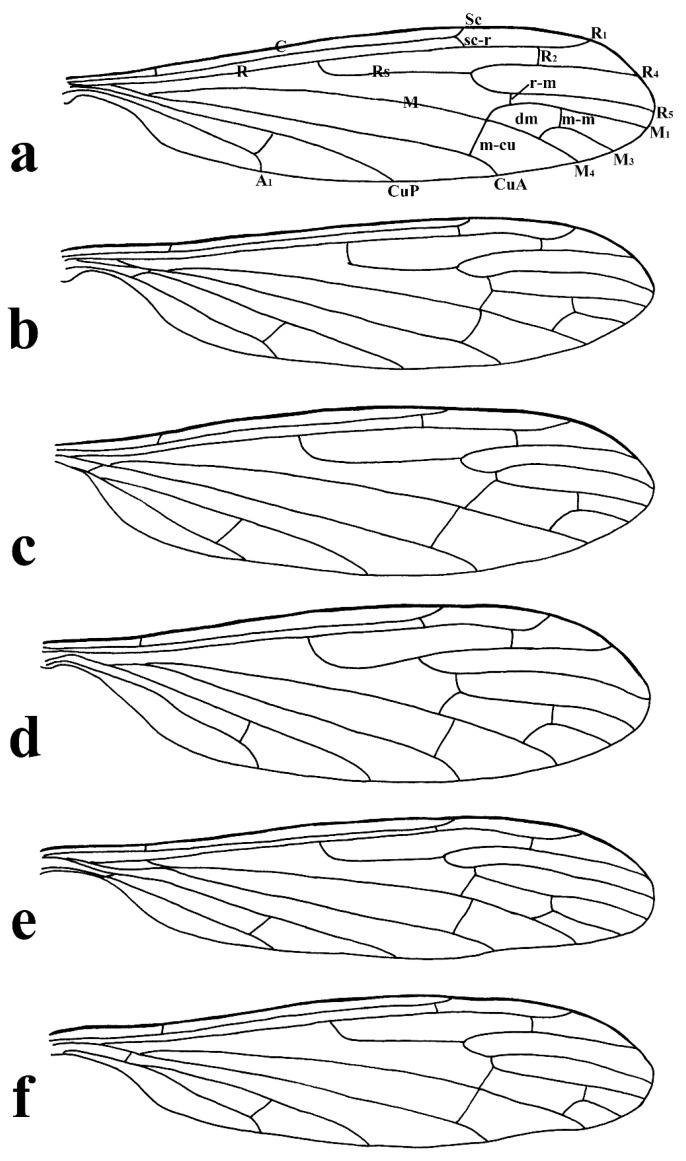
Wing venations of *Discobola* species from China. (**a**) *Discobola acurostris* (Alexander, 1943); (**b**) *Discobola annulata* (Linnaeus, 1758); (**c**) *Discobola armorica* (Alexander, 1942); (**d**) *Discobola margarita* Alexander, 1924; (**e**) *Discobola parvispinula* (Alexander, 1947); (**f**) *Discobola taivanella* (Alexander, 1930).

This study presents detailed accounts of six *Discobola* species from China, including one species newly recorded for the country. Among these, *Discobola acurostris* (Alexander, 1943), a species previously reported from China, is redescribed based on the holotype due to the lack of newly collected material. The remaining five species are redescribed based on recently collected specimens. The six Chinese species of *Discobola* show no significant differences in wing venation (Figure 1); however, variations in wing pigmentation patterns can be used to distinguish species. This study documents a broader range of intraspecific variation in wing markings, which may aid in refining species boundaries. More comprehensive morphological details, particularly of the hypopygial structures, are also provided to support future taxonomic and phylogenetic research.

#### 3.1.1. *Discobola acurostris* (Alexander, 1943)

*Limonia acurostris* Alexander, 1943: 177 [19]. Type locality: China: Sichuan, Mount Emei.

**Figure 2:**
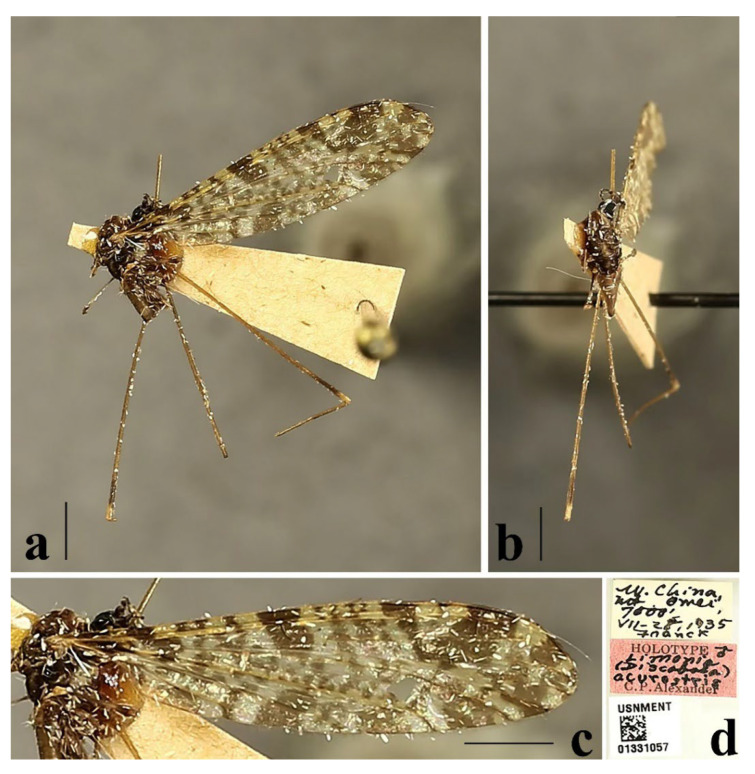
*Discobola acurostris* (Alexander, 1943). (**a**) Habitus of male, lateral view; (**b**) habitus of male, dorsal view; (**c**) wing; (**d**) label data of holotype. Scale bars: 2.0 mm (**a**,**b**); 1.5 mm (**c**). (Photo by Qifei Liu).

Redescription. Wing (Figure 1a and Figure 2) length 9.0 mm. Wing without usual ocellate pattern, instead with large dark brown areas as follows: base section of wing, origin of Rs, sc-r, R_2_ and subapical of the wing tip; cell a_1_, cup, m_4_. Wing with many dark brown spots almost in base 1/4 and middle area of the wing; sometimes spots connecting into a line. Veins yellow, darker in pattern areas. Venation: Sc and sc-r shortly before fork of Rs; tip of R_1_ almost straight. Halter with stem mostly dark brown, base and tip yellow; knob dark brown, tip of knob yellow.

Specimens examined. Holotype, male (USNM), China: Sichuan Province, Mount Emei, Chu Lao Tong Temple (2133.6 m), 28 July 1935, Franck.

Distribution. China (Sichuan).

Remarks. Only the holotype without most of the abdomen was available during this study. A more detailed description of the wing is provided herein. For description and illustration of this species, also see Alexander (1943) [19].

#### 3.1.2. *Discobola annulata* (Linnaeus, 1758)

*Tipula annulata* Linnaeus, 1758: 586 [11]. Type locality: Europe.

*Limnobia imperialis* Loew, 1851: 403 [65]. Type locality: USSR: Leningrad Region.

Redescription. Male. Body length 8.2–8.7 mm, wing length 8.2–8.9 mm.

**Figure 3:**
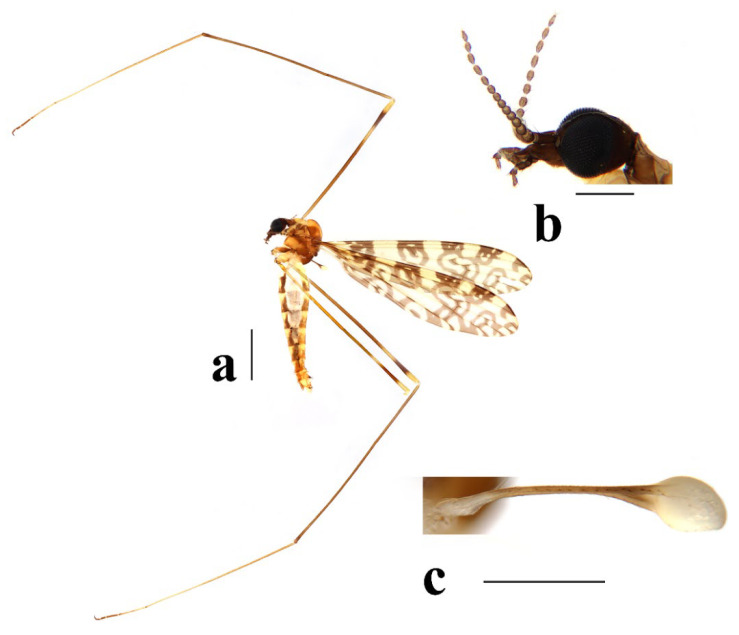
*Discobola annulata* (Linnaeus, 1758). (**a**) Habitus of male, lateral view; (**b**) head, lateral view; (**c**) halter. Scale bars: 2.0 mm (**a**); 0.5 mm (**b**,**c**).

Head (Figure 3a,b) dark brown. Setae on head brown. Antenna dark brown, flagellomeres with short pale apical pedicel. Scape cylindrical, twice as long as wide. Pedicel oval. Basal flagellomeres oval; outer flagellomeres long-oval, tapering apically. Setae on antenna dark brown; setae on each flagellomere shorter than corresponding flagellomere. Rostrum dark brown with dark brown setae. Palpus dark brown with last two segments slightly paler. Palpomeres cylindrical with last segment long-oval; third segment shortest, remaining segments almost equal in length. Setae on palpus dark brown.

**Figure 4:**
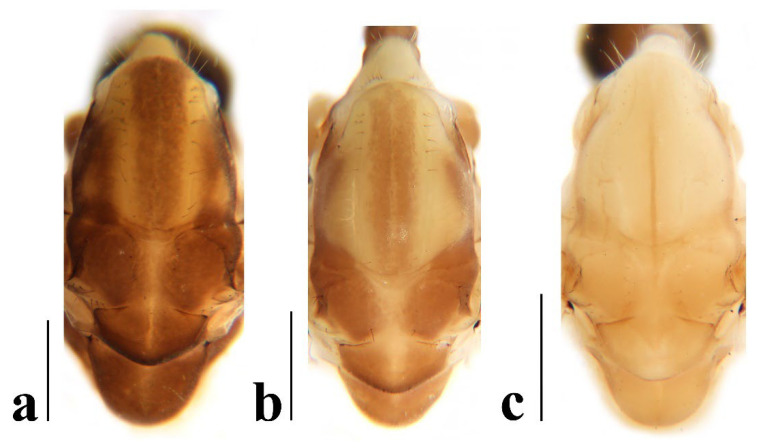
*Discobola annulata* (Linnaeus, 1758). (**a**–**c**) Variations of thorax, dorsal view. Scale bars: 0.5 mm (**a**–**c**).

**Figure 5:**
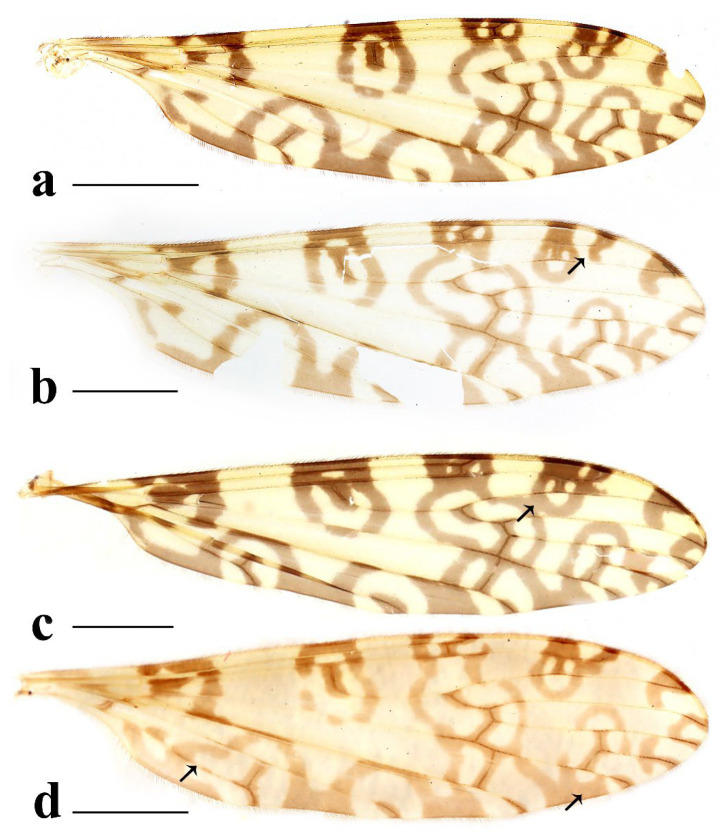
*Discobola annulata* (Linnaeus, 1758). (**a**–**d**) Variations of wing. The black arrows indicate the variable wing spots. Scale bars: 1.5 mm (**a**–**d**).

Thorax (Figure 3a and Figure 4). Pronotum yellow (Figure 4a), pale yellow in some specimens (Figure 4b,c). Prescutum and presutural scutum yellow with three broad dark brown stripes, median stripe longest and broadest, each lateral stripe with a yellow dot in the middle (Figure 4a); prescutum and presutural scutum in some specimens with two brown stripes in the middle and two brown spots on both sides (Figure 4b), or only with one yellowish brown line in the middle (Figure 4c). Postsutural scutum yellow, each lobe with one dark brown spot, and posterior margin yellow (Figure 4a); postsutural scutum in some specimens pale yellow, each lobe with one brown spot, and posterior margin yellow (Figure 4b), or each lobe with one yellow spot, and posterior margin pale yellow (Figure 4c). Scutellum dark brown with one yellow line in the middle, posterior margin brownish black (Figure 4a); scutellum in some specimens brown with middle area yellow anteriorly, and dark brown posteriorly (Figure 4b), or yellow with middle area pale yellow posteriorly (Figure 4c). Mediotergite dark brown (Figure 4a), brown or pale yellow in some specimens (Figure 4b,c). Pleuron (Figure 3a) yellow, with three brown stripes. Setae on thorax dark brown. Fore and mid coxae dark brown, hind coxa yellow; trochanters yellow; femora yellow basally and gradually darkened to brown, tip with two clear yellow rings, enclosing a broad dark brown ring; tibiae brown; first tarsus brown to yellowish brown, remaining tarsi yellowish brown basally and gradually darkened to brown. Setae on legs brown. Wing (Figure 1b and Figure 5) yellow (Figure 5a,c,d), pale yellow in some specimens (Figure 5b); with usual brown ocellate pattern as follows: base of wing, base of Rs, sc-r, R_2_, tip of wing, fork of R_4+5_, r-m, m-m, m-cu, tip of CuP, tip of A_1_ (Figure 5a). Ocellate pattern in R_2_ sometimes separated (Figure 5b); ocellate pattern in R_2_ and in r-m sometimes connected (Figure 5c); ocellate pattern in m-m and in tip of A_1_ sometimes enlarged (Figure 5d). Veins brown, darker in pattern areas. Venation: Sc beyond fork of Rs; tip of R_1_ slightly curved; basal section of M_3_ about 1.5–2 times length of m-m; r-m about 2/5–2/3 length of inner edge of dm, m-cu at or beyond fork of M. Halter brown with base of stem and knob yellow (Figure 3a,c).

Abdomen (Figure 3a). Tergite 1 yellow, tergite 2 yellow with lateral and caudal area brown, tergites 3–8 brown with basal 1/3 yellow. Sternites 1–8 yellow with lateral and caudal 1/3 dark brown. Segment 9 yellow. Setae on abdomen brown.

**Figure 6:**
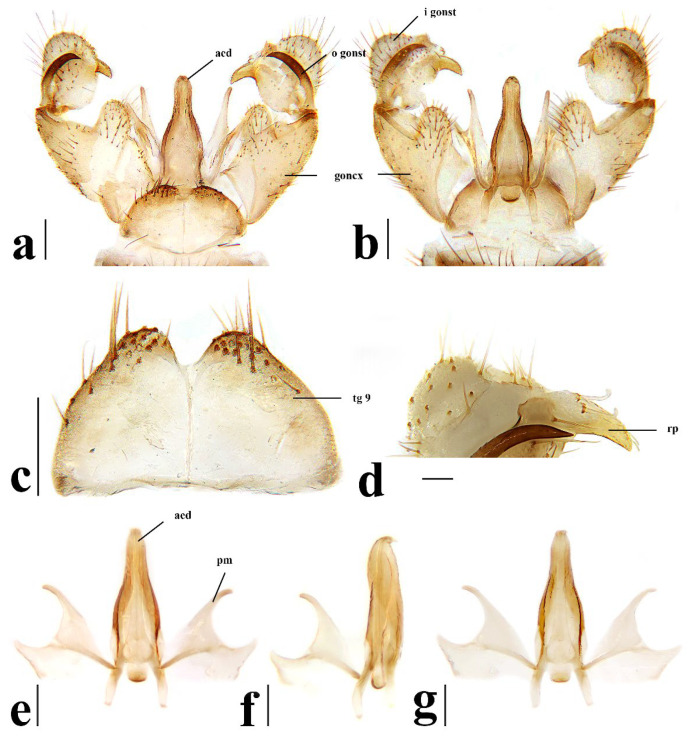
*Discobola annulata* (Linnaeus, 1758). (**a**) Male hypopygium, dorsal view; (**b**) male hypopygium, ventral view; (**c**) tergite 9, dorsal view; (**d**) rostral prolongation, dorsal view; (**e**) complex of aedeagus, dorsal view; (**f**) complex of aedeagus, lateral view; (**g**) complex of aedeagus, ventral view. Scale bars: 0.2 mm (**a**–**c**,**e**–**g**); 0.05 mm (**d**).

Hypopygium (Figure 6) yellowish brown. Tergite 9 (Figure 6a,c) trapeziform, posterior margin with U-shaped notch; each lobe with about 20 long brown setae. Gonocoxite (Figure 6a,b) cylindrical, middle with an elongated ventromesal lobe; gonocoxite with short brown setae, ventromesal lobe with dense short brown setae. Outer gonostylus (Figure 6a,b) narrow, arched with tip acute and sclerotized. Inner gonostylus (Figure 6a,b) as long as outer gonostylus, oval. Rostral prolongation (Figure 6a,b,d) short, bent, near base bearing tubercles armed with two separated curved spines. Paramere (Figure 6e–g) finger-shaped, wide at base, narrowed and curved outward apically. Aedeagus (Figure 6a,b,e–g) cylindrical, widened at base, slightly narrowed at middle.

**Figure 7:**
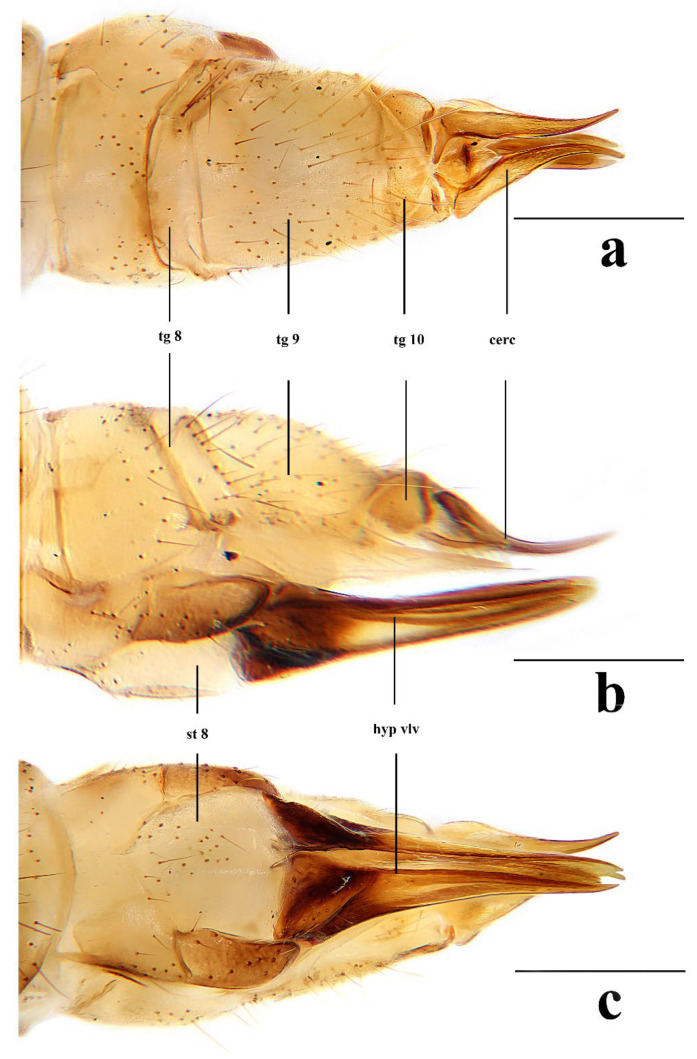
*Discobola annulata* (Linnaeus, 1758). (**a**) Female ovipositor, dorsal view; (**b**) female ovipositor, lateral view; (**c**) female ovipositor, ventral view. Scale bars: 0.5 mm (**a**–**c**).

Female. Body length 7.0–9.5 mm, wing length 7.5–10.0 mm. Generally similar to male by body coloration. Ovipositor (Figure 7) with tergite 9 yellow. Tergite 10 yellowish brown, slightly darker in caudal margin. Cercus short, yellowish brown with tip darker (Figure 7a,b). Hypogynial valve brown, with 1/3 brownish black at base and paler at middle (Figure 7b,c).

Specimens examined. 1 female, China: Gansu Province, Diebu County, Duoer Forest Farm, Houxizanggou (2733 m), 18 August 2016, Liang Wang. 1 female, China: Guangxi Autonomous Region, Longsheng county, Huaping National Nature Reserve, Anjiangping, 18 June 2021 (flight interception trap). 1 male 1 female, China: Hunan Province, Sangzhi County, Mount Doupeng (1710 m), 1 August 2015, Yuqiang Xi. 1 female, China: Hunan Province, Sangzhi County, Mount Tianping (1500 m), 18 June 2014, Xiao Zhang. 1 male, China: Yunnan Province, Gongshan County, 5 May 2018, Liang Wang (light trap). 1 male, China: Yunnan Province, Nanjian County, Mount Wuliang (2221 m), 18 July 2016, Qicheng Yang.

Distribution. China (Gansu, Guangxi, Hunan, Yunnan, Taiwan); Canada; USA; Austria; Bosnia; Bulgaria; Czech Rep.; Estonia; Finland; France; Germany; Great Britain; Italy; Latvia; Lithuania; Norway; Poland; Romania; Slovakia; Slovenia; Sweden; Switzerland; Ukraine; Russia; Kazakhstan; Mongolia; Korea; Japan; India; Malaysia; Nepa; Philippines; New Guinea.

#### 3.1.3. *Discobola armorica* (Alexander, 1942)

*Limonia armorica* Alexander, 1942: 54 [17]. Type locality: Burma: Adung Valley.

Redescription. Male. Body length 11.3 mm, wing length 11.5 mm.

**Figure 8:**
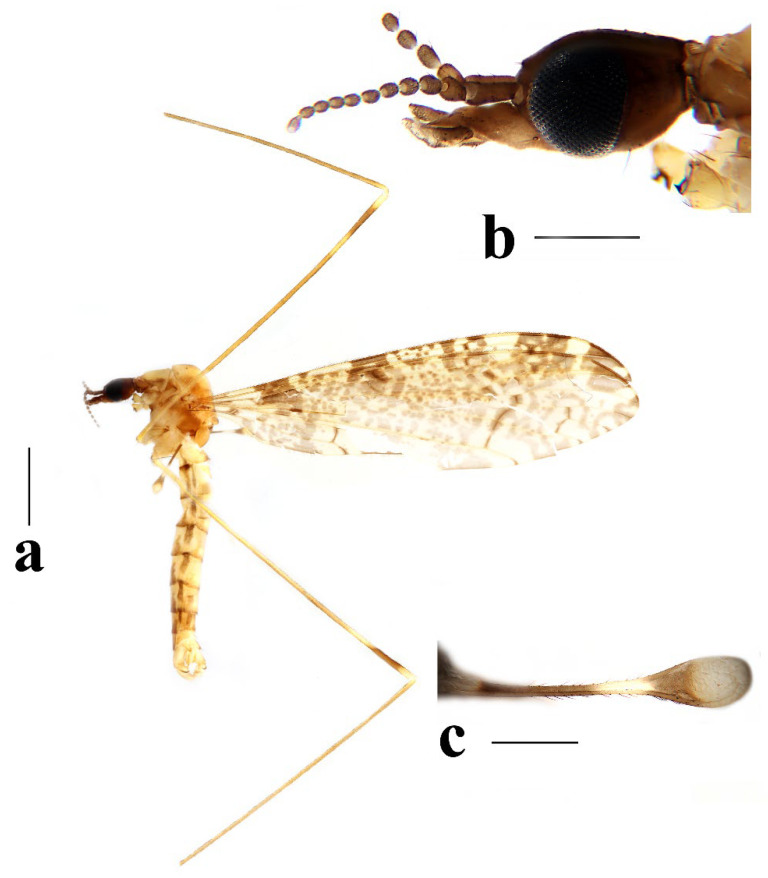
*Discobola armorica* (Alexander, 1942). (**a**) Habitus of male, lateral view; (**b**) head, lateral view; (**c**) halter. Scale bars: 2.0 mm (**a**); 0.5 mm (**b**,**c**).

Head (Figure 8a,b) dark brown. Setae on head brown. Antenna dark brown, flagellomeres with short pale apical pedicel. Scape cylindrical, 2.5 times as long as wide. Pedicel oval. Basal flagellomeres oval, tip flat; outer flagellomeres long-oval, tapering apically. Setae on antenna dark brown, setae on each flagellomere shorter than corresponding flagellomere. Rostrum brown with dark-brown setae. Palpus dark brown. Palpomeres cylindrical with last segment long-oval; third segment shortest, remaining segments almost equal in length. Setae on palpus dark brown.

**Figure 9:**
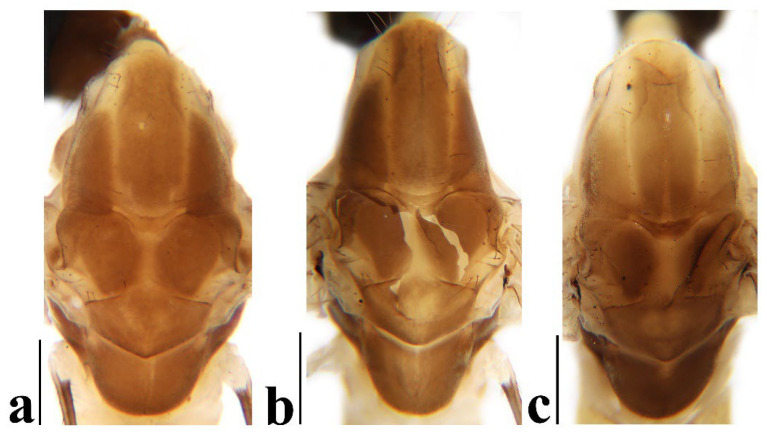
*Discobola armorica* (Alexander, 1942). (**a**–**c**) Variations of thorax, dorsal view. Scale bars: 0.5 mm (**a**–**c**).

**Figure 10:**
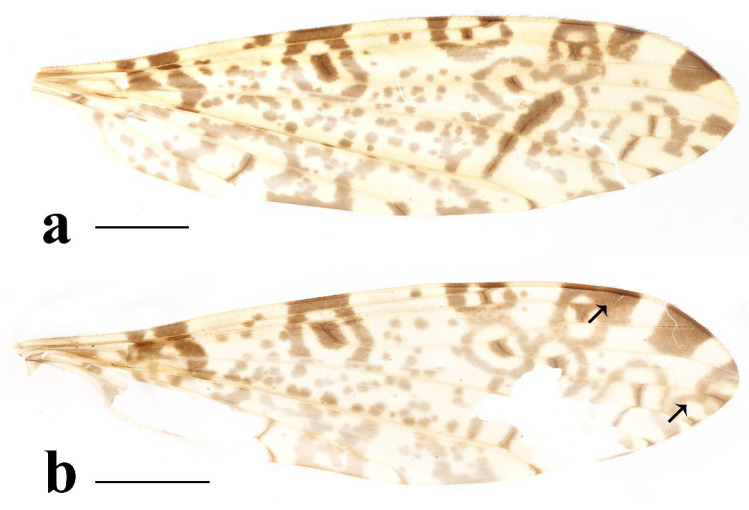
*Discobola armorica* (Alexander, 1942). (**a**,**b**) Variations of wing. The black arrows indicate the variable wing spots. Scale bars: 1.5 mm (**a**,**b**).

Thorax (Figure 8a and Figure 9). Pronotum yellow (Figure 9a). Prescutum and presutural scutum yellow with three broad brown stripes, the median stripe longest and broadest; lateral stripes extending onto lobes of postsutural scutum (Figure 9a,b), some specimens with paler anterior (Figure 9c). Postsutural scutum yellow, each lobe with a brown spot, and posterior margin pale yellow (Figure 9a); postsutural scutum in some specimens pale yellow, each lobe with a dark brown spot, and posterior margin pale yellow (Figure 9b,c). Scutellum brown (Figure 9a); scutellum in some specimens brown or dark brown with middle area yellow (Figure 9b,c). Mediotergite brown (Figure 9a,b) or dark brown (Figure 9c). Pleuron (Figure 8a) yellow with one broad longitudinal brown stripe. Setae on thorax dark brown. Coxae and trochanters yellow; femora yellow basally and gradually darkened to brown, tip with two clear yellow rings, enclosing a broad dark brown ring; tibiae yellowish brown. Setae on legs dark brown. Wing (Figure 1c and Figure 10) pale brown, with usual brown ocellate pattern as follows: base of wing, base of Rs, sc-r, R_2_, fork of R_4+5_, cord, m-m, tip of M_3_, tip of CuP, tip of A_1_ (Figure 10a); ocellate pattern in tip of M_3_ sometimes enlarged (Figure 10b). Wing with two large spots at tip of R_1_ and tip of R_4_ (Figure 10a), sometimes large spot at tip of R_1_ connected to the ocellate pattern in R_2_ (Figure 10b). Wing also with many brown spots in cell r_1_, cell r, m, dm, cua, and a_1_. Veins brown, darker in pattern areas. Venation: Sc ending slightly before or at fork of Rs; tip of R_1_ slightly curved; basal section of M_3_ about twice length of m-m; r-m about 1/6 length of inner edge of dm; m-cu before fork of M. Stem of halter dark brown, base and tip yellow; knob pale white, base brown (Figure 8a,c).

Abdomen (Figure 8a). Tergite 1 yellow with caudal edge brown, tergite 2 yellow with caudal 1/3 brown, tergites 3–7 yellow with caudal edge brown, tergite 8 yellow. Sternite 1 yellow, sternites 2–7 yellow with lateral and caudal area brown, sternite 8 brown. Setae on abdomen brown.

**Figure 11:**
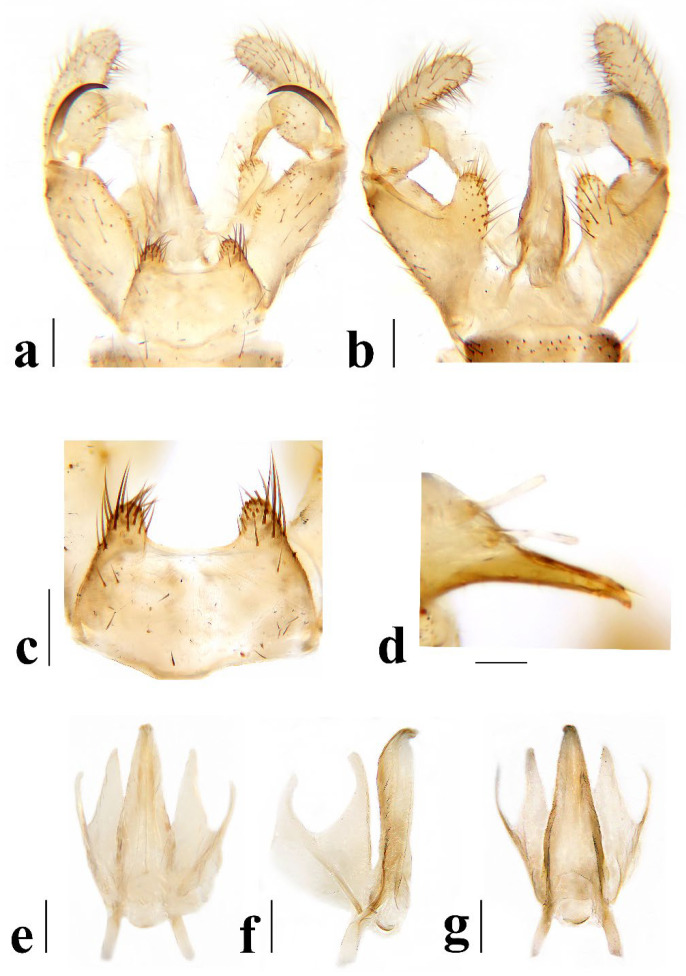
*Discobola armorica* (Alexander, 1942). (**a**) Male hypopygium, dorsal view; (**b**) male hypopygium, ventral view; (**c**) tergite 9, dorsal view; (**d**) rostral prolongation, dorsal view; (**e**) complex of aedeagus, dorsal view; (**f**) complex of aedeagus, lateral view; (**g**) complex of aedeagus, ventral view. Scale bars: 0.2 mm (**a**–**c**,**e**–**g**); 0.05 mm (**d**).

Hypopygium (Figure 11) yellow. Tergite 9 (Figure 11a,c) trapeziform, posterior margin with broad U-shaped notch; each lobe with about 20 long brown setae. Gonocoxite (Figure 11a,b) cylindrical, middle with an elongated ventromesal lobe; gonocoxite with brown setae, ventromesal lobe with dense brown setae. Outer gonostylus (Figure 11a,b) narrow, arched with tip acute and sclerotized. Inner gonostylus (Figure 11a,b) about 1.5 times length of outer gonostylus, long-oval, base with a spherical expansion. Rostral prolongation (Figure 11d) short and slender, near base bearing tubercles armed with two separated spines. Paramere (Figure 11e–g) finger-shaped, wide at base, narrowed and curved outward apically. Aedeagus (Figure 11a,b,e–g) cylindrical, widened at base, slightly narrowed at middle.

**Figure 12:**
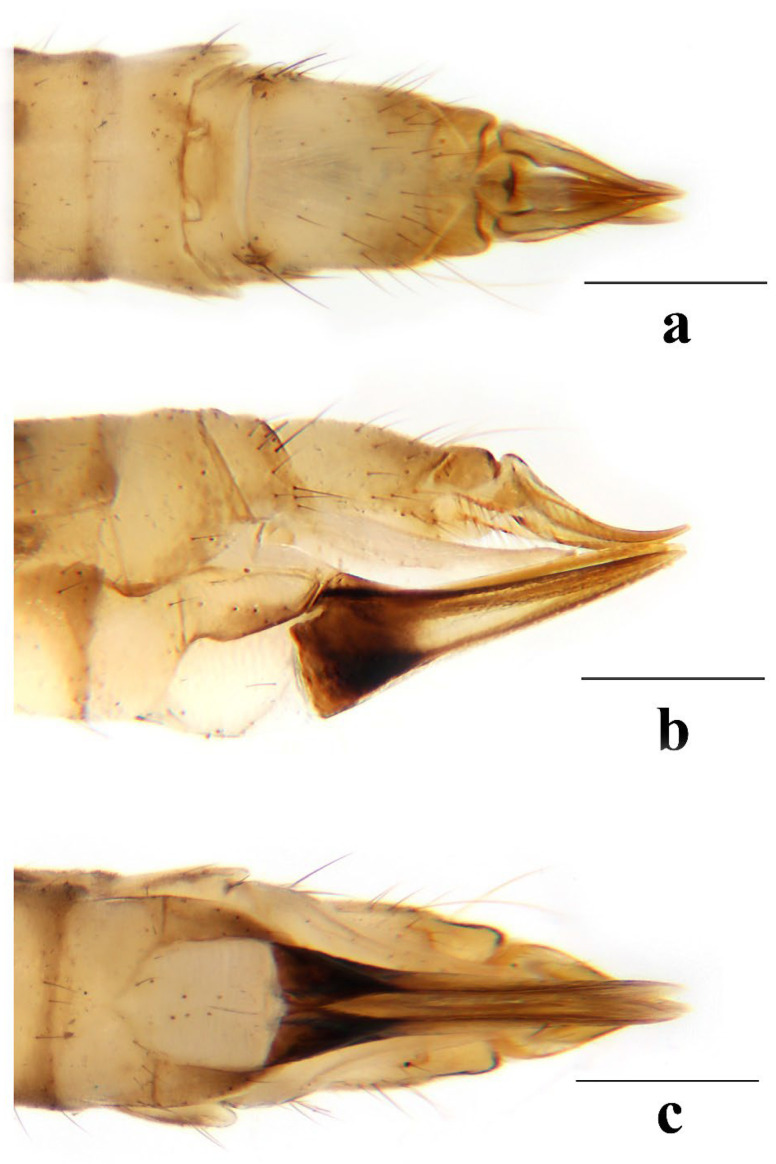
*Discobola armorica* (Alexander, 1942). (**a**) Female ovipositor, dorsal view; (**b**) female ovipositor, lateral view; (**c**) female ovipositor, ventral view. Scale bars: 0.5 mm (**a**–**c**).

Female. Body length 8.5–10.7 mm, wing length 9.5–11.3 mm. Generally similar to male by body coloration. Ovipositor (Figure 12) with tergite 9 yellow, slightly darker in caudal margin. Tergite 10 yellowish brown. Cercus short, yellowish brown with tip darker (Figure 12a,b). Hypogynial valve brown, with 2/5 brownish black at base and paler at middle (Figure 12b,c).

Specimens examined. Paratype, 1 male, Myanmar, Adung Valley (3657.6 m), 16 August 1931, Kingdon-Ward and Lord Cranbrook. Other materials: 1 female, China: Sichuan Province, Pingwu County, Wanglang National Nature Reserve, Great Lawn (2930 m), 3 August 2016, Yuqiang Xi. 1 male 1 female, China: Xizang Autonomous Region, Bomi County, Bagai Township (3045 m), 2 July 2018, Liang Wang.

Distribution. China (Sichuan, Xizang); Myanmar.

#### 3.1.4. *Discobola margarita* Alexander, 1924

*Discobola margarita* Alexander, 1924: 539 [12]. Type locality: Japan: Hokkaidō, Kamiotoineppu.

Redescription. Male. Body length 6.8–8.8 mm, wing length 6.7–8.5 mm.

**Figure 13:**
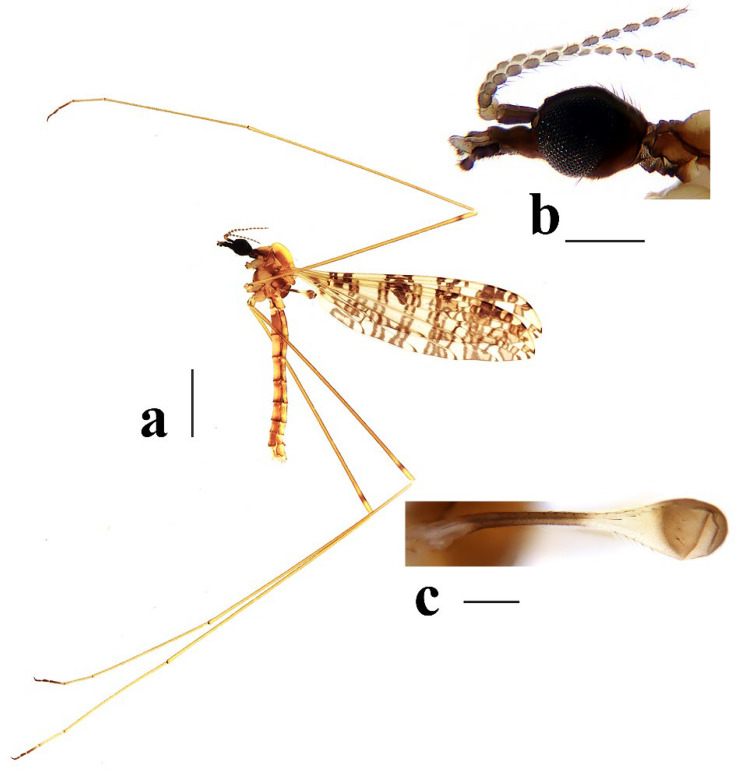
*Discobola margarita* Alexander, 1924. (**a**) Habitus of male, lateral view; (**b**) head, lateral view; (**c**) halter. Scale bars: 2.0 mm (**a**); 0.5 mm (**b**,**c**).

Head (Figure 13a,b) dark brown. Setae on head dark brown. Antenna dark brown with pedicel yellow, flagellomeres with short pale apical pedicel. Scape cylindrical, three times as long as wide. Pedicel oval. Basal flagellomeres oval, tip flat; outer flagellomeres long-oval, tapering apically. Setae on antenna dark brown, setae on each flagellomere shorter than corresponding flagellomere. Rostrum dark brown with dark brown setae. Palpus dark brown with last segment paler. Palpomeres cylindrical with last segment long-oval; third segment shortest, remaining segments almost equal in length. Setae on palpus dark brown.

**Figure 14:**
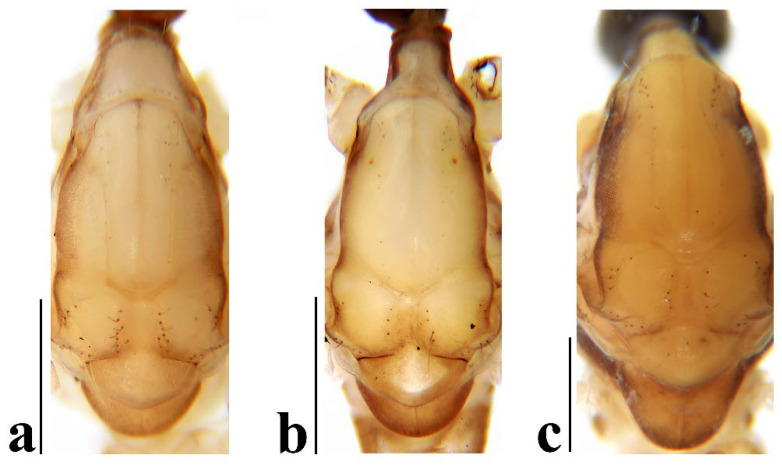
*Discobola margarita* Alexander, 1924. (**a**–**c**) Variations of thorax, dorsal view. Scale bars: 0.5 mm (**a**–**c**).

**Figure 15:**
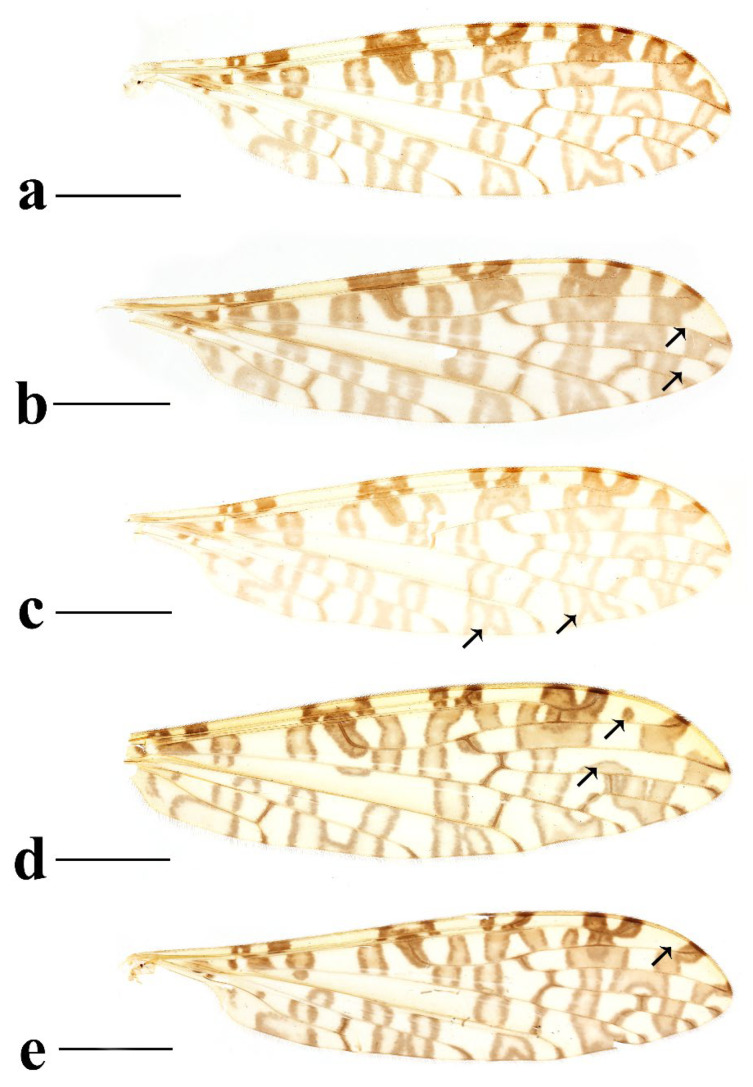
*Discobola margarita* Alexander, 1924. (**a**–**e**) Variations of wing. The black arrows indicate the variable wing spots. Scale bars: 1.5 mm (**a**–**e**).

Thorax (Figure 13a and Figure 14). Pronotum yellow with lateral margin brown. Prescutum and presutural scutum yellow with lateral margin brown. Postsutural scutum pale yellow (Figure 14a,b) or yellow (Figure 14c) with lateral margin dark brown. Scutellum uniformly yellow (Figure 14a,c) or yellow with middle area pale yellow (Figure 14b). Mediotergite yellowish brown with posterior margin brown. Pleuron (Figure 13a) yellowish brown with two longitudinal brown stripes. Setae on thorax brown. Coxae and trochanters yellow; femora yellowish brown, tip with two clear yellow rings, enclosing a narrow dark brown ring; tibiae yellow; first and second tarsi yellow, remaining tarsi brown. Setae on legs brown. Wing (Figure 1d and Figure 15) pale yellow with irregular ocellate pattern, ocellate pattern bicolored with central area paler yellow and margin dark brown; ocellate pattern as follows: base of wing, base of Rs, sc-r, R_2_, tip of R_4_, cord, m-m, tip of CuP, tip of A_1_ (Figure 15a); ocellate pattern in tip of R_4_ sometimes reduced and separated into two (Figure 15b,c,e) or three spots (Figure 15d); ocellate pattern in m-m sometimes reduced (Figure 15b,c,e); spot in tip of cell cua sometimes separated into two spots (Figure 15c,d). Veins brown, darker in the tip of all veins. Venation: Sc ending beyond fork of Rs; tip of R_1_ strongly curved; basal section of M_3_ about twice length of m-m; r-m about 1/2 length of inner edge of dm; m-cu slightly beyond fork of M or at 1/6–1/4 length of cell dm. Stem of halter dark brown, base and tip yellow; knob brown, base yellow (Figure 13a,c).

Abdomen (Figure 13a). Segments 1–8 yellow with caudal margins brown. Setae on abdomen brown.

**Figure 16:**
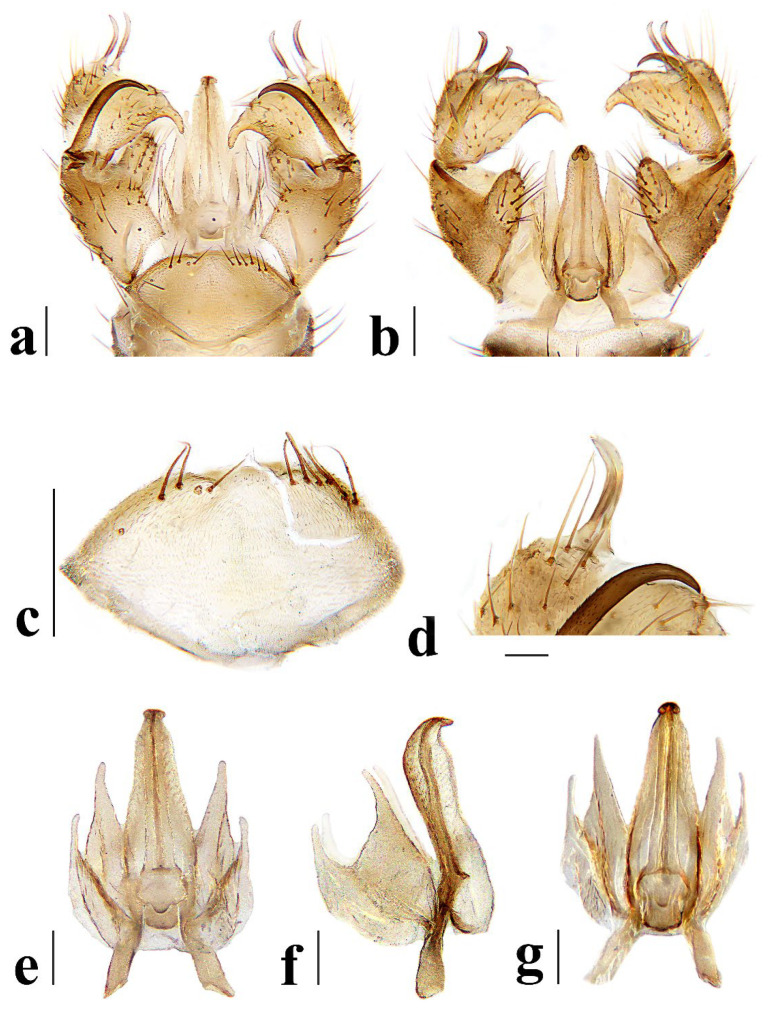
*Discobola margarita* Alexander, 1924. (**a**) Male hypopygium, dorsal view; (**b**) male hypopygium, ventral view; (**c**) tergite 9, dorsal view; (**d**) inner gonostylus, dorsal view; (**e**) complex of aedeagus, dorsal view; (**f**) complex of aedeagus, lateral view; (**g**) complex of aedeagus, ventral view. Scale bars: 0.2 mm (**a**–**c**,**e**–**g**); 0.05 mm (**d**).

Hypopygium (Figure 16) yellow. Tergite 9 (Figure 16a,c) nearly oval-shaped, transverse, posterior margin with indistinct emargination medially; posterior margin with six setae each side. Gonocoxite (Figure 16a,b) cylindrical, middle with an elongated ventromesal lobe; gonocoxite with long brown setae, ventromesal lobe with dense long brown setae. Outer gonostylus (Figure 16a,b) narrow, arched with tip acute and sclerotized. Inner gonostylus (Figure 16a,b,d) slightly shorter than outer gonostylus, oval, with two long and stout setae at tip. Rostral prolongation short, bent, with several short setae at apex. Paramere (Figure 16e–g) finger-shaped, wide at base, narrowed apically, curved outward. Aedeagus (Figure 16a,b,e–g) cylindrical, widened at base, slightly narrowed at middle.

**Figure 17:**
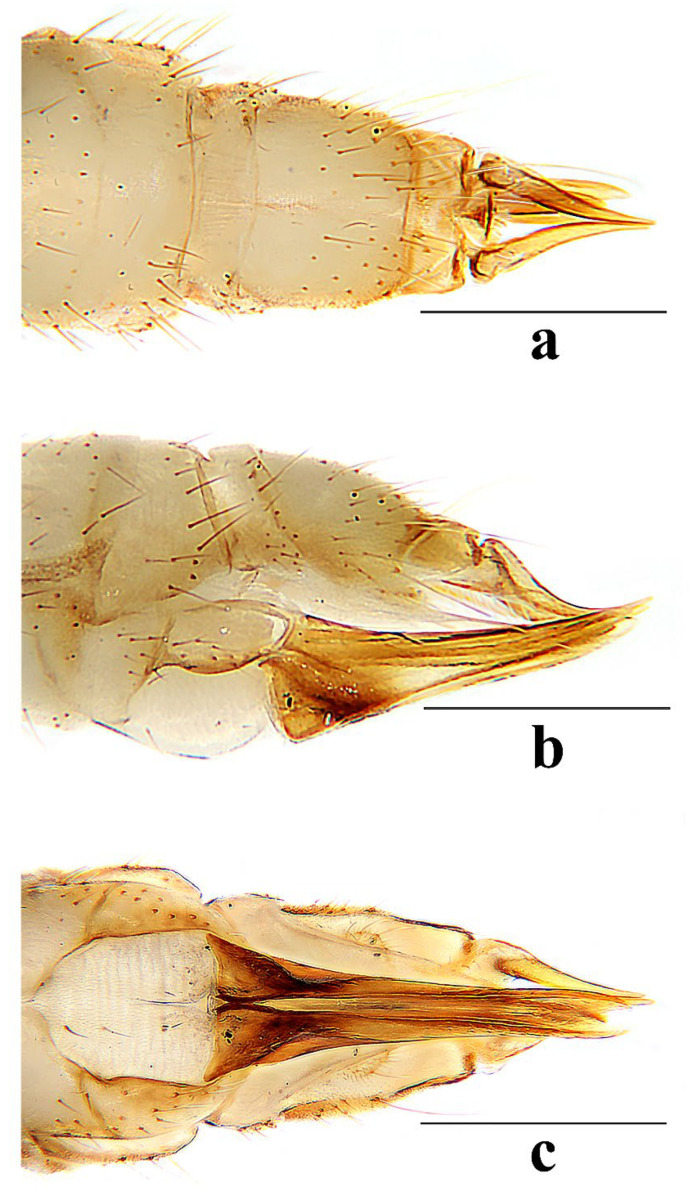
*Discobola margarita* Alexander, 1924. (**a**) Female ovipositor, dorsal view; (**b**) female ovipositor, lateral view; (**c**) female ovipositor, ventral view. Scale bars: 0.5 mm (**a**–**c**).

Female. Body length 6.1–8.7 mm, wing length 6.0–8.5 mm. Generally similar to male by body coloration. Ovipositor (Figure 17) with tergite 9 pale yellow. Tergite 10 yellow, slightly darker in caudal margin. Cercus short, yellowish brown with tip darker (Figure 17a,b). Hypogynial valve brown, with 1/3 brown at base and paler at middle (Figure 17b,c).

Specimens examined. 1 male, China: Fujian Province, Tianbaoyan National Nature Reserve, Xiyang Management Station (700 m), 29 April 2015 (Malaise trap). 1 female, China: Guangxi Autonomous Region, Longsheng county, Huaping National Nature Reserve, Anjiangping, 18 June 2021 (flight interception trap). 1 female, China: Hebei Province, Xinglong County, Dagou Village (860 m), 11 June 2014 to 11 November 2014 (Malaise trap). 1 female, China: Hunan Province, Sangzhi County, Mount Doupeng, 6 August 2015, Yuqiang Xi (Malaise trap). 1 female, China: Liaoning Province, Xinbin County, Douling Forest Farm, 12 August 2009, Maoling Sheng. 1 female, China: Liaoning Province, Shenyang Botanical Garden, 8 August 2016, Yan Li (Malaise trap). 1 male, China: Shaanxi Province, Feng County, near Tongtianhe National Forest Park (1551.5 m), 25 June 2024, Hanhuiying Lv. 1 female, China: Taiwan Province, Jiayi Country, Peizaitong Forest Road (1100 m), 7 June 2012, Lihua Wang (light trap). 1 female, China: Taiwan Province, Jiayi Country, Peizaitong Forest Road (1100 m), 7 June 2012, Lihua Wang (light trap). 3 males 2 females, China: Taiwan Province, Yilan Country, Yuanshan Township, Fushan Botanical Garden (635 m), 12 September 2010, Ding Yang. 2 males, China: Yunnan Province, Gongshan County, 5 May 2018, Liang Wang (light trap). 1 male, China: Zhejiang Province, Linan District, Qingliangfeng National Nature Reserve, Qianqingtang, 11 October 2012 (Malaise trap).

Distribution. China (Fujian, Guangxi, Hebei, Hunan, Liaoning, Shaanxi, Taiwan, Yunnan, Zhejiang); Russia; Korea; Japan; India; Thailand.

#### 3.1.5. *Discobola parvispinula* (Alexander, 1947)

*Limonia* (*Discobola*) *parvispinula* Alexander,1947: 350 [20]. Type locality: Japan: Honshu, Kamikochi.

Redescription. Male. Body length 12.4 mm, wing length 9.5 mm.

**Figure 18:**
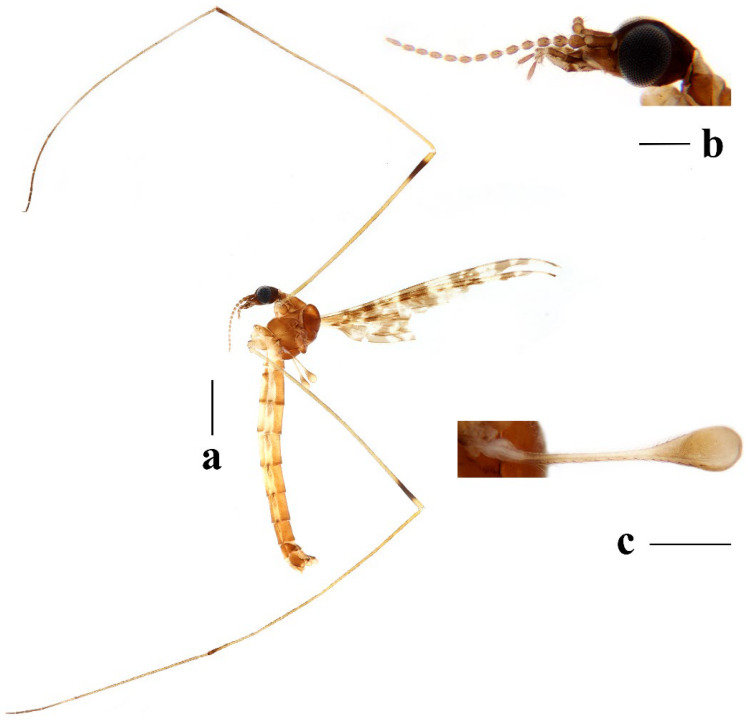
*Discobola parvispinula* (Alexander, 1947). (**a**) Habitus of male, lateral view; (**b**) head, lateral view; (**c**) halter. Scale bars: 2.0 mm (**a**); 0.5 mm (**b**,**c**).

Head (Figure 18a,b) dark brown. Setae on head dark brown. Antenna brown, with scape dark brown, flagellomeres with short pale apical pedicel. Scape cylindrical, 2.5 times as long as wide. Pedicel oval. Flagellomeres oval, last flagellomere long-oval. Setae on antenna dark brown, setae on each flagellomere slightly shorter than corresponding flagellomere. Rostrum dark brown with dark brown setae. Palpus dark brown with third segment paler. Palpomeres cylindrical with last segment long-oval; third segment shortest, remaining segments almost equal in length. Setae on palpus dark brown.

**Figure 19:**
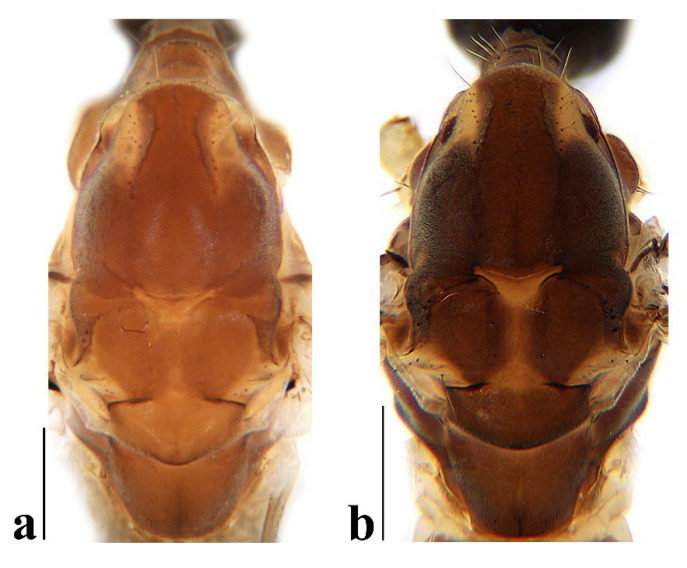
*Discobola parvispinula* (Alexander, 1947). (**a**,**b**) Variations of thorax, dorsal view. Scale bars: 0.5 mm (**a**,**b**).

**Figure 20:**
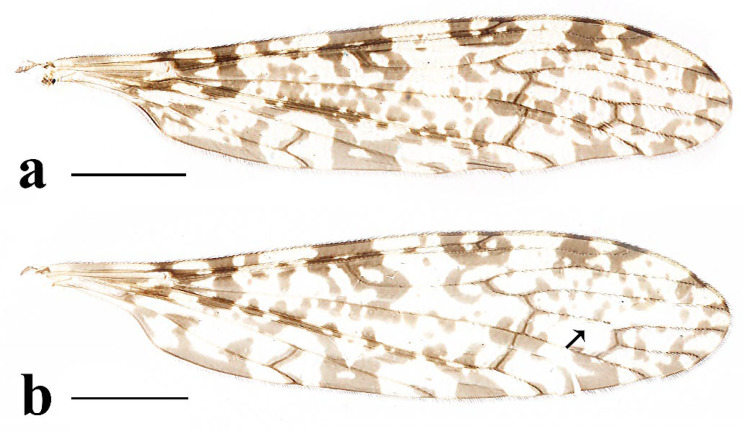
*Discobola parvispinula* (Alexander, 1947). (**a**,**b**) Variations of wing. The black arrow indicates the variable wing spot. Scale bars: 1.5 mm (**a**,**b**).

Thorax (Figure 18a and Figure 19). Pronotum brown (Figure 19). Prescutum and presutural scutum yellow with three broad brown stripes fused in the middle, median stripe longest and broadest, and lateral stripes extending onto lobes of postsutural scutum. Postsutural scutum yellow, each lobe with one brown spot, brown spots fused in the middle. Scutellum brown, with anterior and posterior margin darker. Mediotergite brown (Figure 18a). Some specimens with a darker thorax overall and the postsutural scutum with middle area yellow (Figure 19b). Pleuron (Figure 18a) brown, with dorsal half of anepimeron and katepisternum paler. Setae on thorax yellow. Coxae and trochanters yellow; femora yellow, with a broad dark brown ring at subtip; tibiae yellow with tip brown; basal tarsus yellow with tip brown, remaining tarsi brown. Setae on legs brown. Wing (Figure 1e and Figure 20) pale yellow, with broken brown ocellate pattern as follows: base of wing, base of Rs, sc-r, R_2_, cord, m-m, tip of CuP, tip of A_1_ (Figure 20a); ocellate pattern in m-m sometimes reduced (Figure 20b). Wing with many brown spots on both sides of cell m, r_4_, and r_5_. Veins brown, darker in pattern areas. Venation: Sc beyond fork of Rs; tip of R_1_ slightly curved; basal section of M_3_ slightly longer than m-m; r-m about 1/4–3/4 length of inner edge of dm; m-cu slightly before or beyond fork of M. Stem of halter yellow with a yellowish brown subbasal ring; knob yellowish brown (Figure 18a,c).

Abdomen (Figure 18a). Tergites 1–7 yellowish brown. Sternites 1–5 yellow, with lateral and caudal area brown. Sternites 6–7 yellowish brown. Segment 8 brown. Setae on abdomen brown.

**Figure 21:**
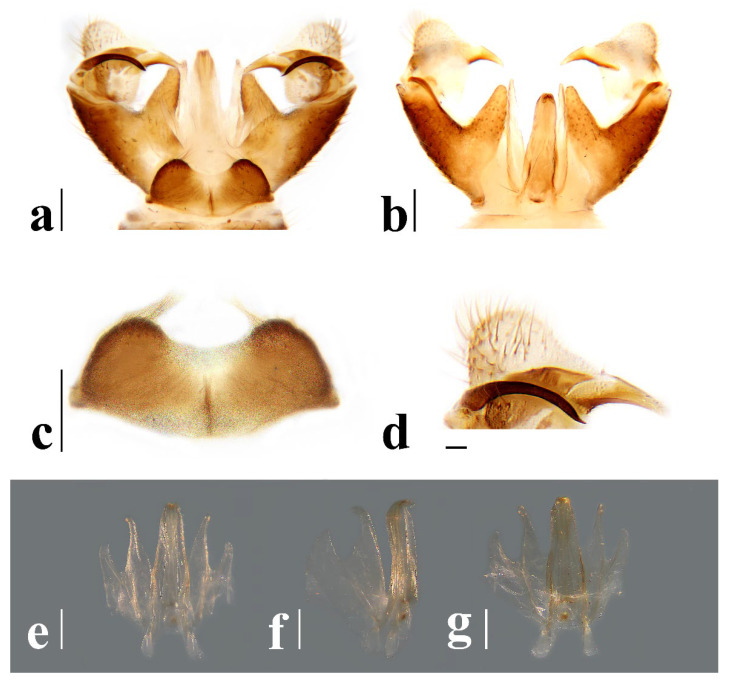
*Discobola parvispinula* (Alexander, 1947). (**a**) Male hypopygium, dorsal view; (**b**) male hypopygium, ventral view; (**c**) tergite 9, dorsal view; (**d**) rostral prolongation, dorsal view; (**e**) complex of aedeagus, dorsal view; (**f**) complex of aedeagus, lateral view; (**g**) complex of aedeagus, ventral view. Scale bars: 0.2 mm (**a**–**c**,**e**–**g**); 0.05 mm (**d**).

Hypopygium (Figure 21) brown. Tergite 9 (Figure 21a,c) trapeziform, transverse, posterior margin with broad U-shaped notch; each lobe with dense, long brown setae. Gonocoxite (Figure 21a,b) cylindrical, basal 1/3 with an elongated, conical ventromesal lobe; gonocoxite with short brown setae, ventromesal lobe with short brown setae. Outer gonostylus (Figure 21a,d) narrow, arched with tip acute and sclerotized. Inner gonostylus (Figure 21a,b) as long as outer gonostylus, oval. Rostral prolongation (Figure 21a,b,d) elongated, bent, near base bearing a very small and peg-shaped spine. Paramere (Figure 21e–g) finger-shaped, wide at base, narrowed and curved apically. Aedeagus (Figure 21a,b,e–g) cylindrical, widened at base, slightly narrowed at middle.

**Figure 22:**
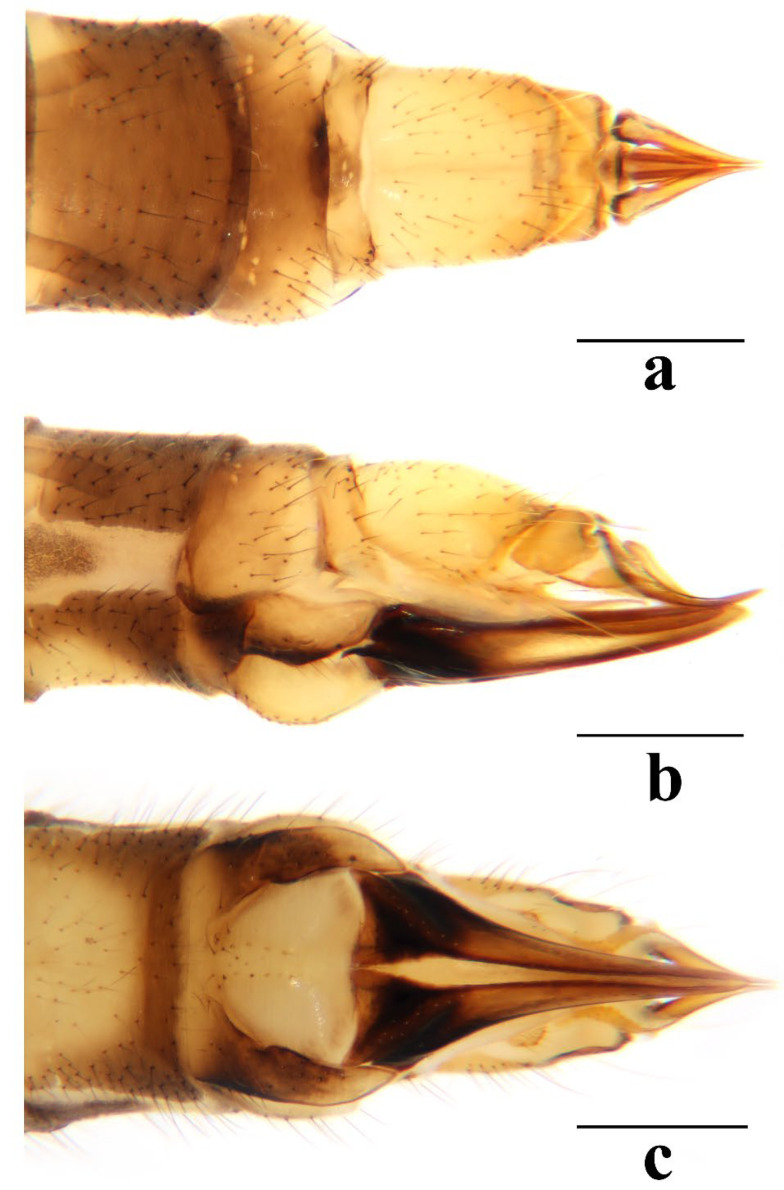
*Discobola parvispinula* (Alexander, 1947). (**a**) Female ovipositor, dorsal view; (**b**) female ovipositor, lateral view; (**c**) female ovipositor, ventral view. Scale bars: 0.5 mm (**a**–**c**).

Female. Body length 11.8 mm, wing length 9.0 mm. Generally similar to male by body coloration. Ovipositor (Figure 22) with tergite 9 pale yellow, slightly darker in caudal margin. Tergite 10 yellow, slightly darker in caudal margin. Cercus short, yellow with base darker (Figure 22a,b). Hypogynial valve brown, with 1/3 brownish black at base (Figure 22b,c).

Specimens examined. 1 female, China: Inner Mongolia Autonomous Region, Genhe County, Genhe Aoluguya Airport (685.2 m), 28 July 2022, Hanhuiying Lv. 1 male, China: Inner Mongolia Autonomous Region, Harqin Banner, Wangyedian National Forest Park (1392–1620 m), 23 August 2014, Li Shi.

Distribution. China (Inner Mongolia); Czech Rep; Lithuania; Poland; Romania; Slovakia; Ukraine; Russia; Kazakhstan; Japan.

Remarks. Although this species was previously reported as widely distributed across the Palaearctic region, its occurrence in China had not been recorded until now. The current finding from Inner Mongolia, China represents the first verified record of this species within the country.

#### 3.1.6. *Discobola taivanella* (Alexander, 1930)

*Limonia taivanella* Alexander, 1930: 511 [15]. Type locality: China: Taiwan, Hassensan.

Redescription. Male. Body length 7.1–10.6 mm, wing length 7.2–11.5 mm.

**Figure 23:**
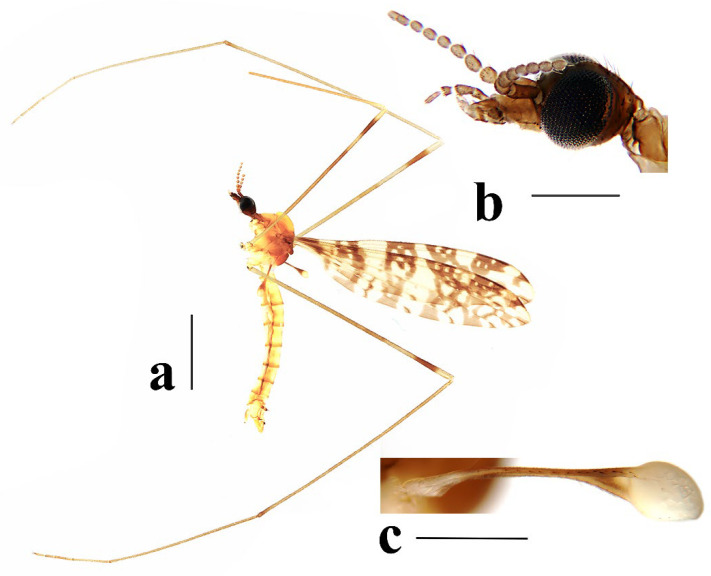
*Discobola taivanella* (Alexander, 1930). (**a**) Habitus of male, lateral view; (**b**) head, lateral view; (**c**) halter. Scale bars: 2.0 mm (**a**); 0.5 mm (**b**,**c**).

Head (Figure 23a,b) dark brown. Setae on head dark brown. Antenna brown, with scape dark brown, flagellomeres with short pale apical pedicel. Scape cylindrical, twice as long as wide. Pedicel oval. Basal flagellomeres oval; outer flagellomeres long-oval, tapering apically. Setae on antenna dark brown, setae on each flagellomere shorter than corresponding flagellomere. Rostrum dark brown with dark brown setae. Palpus dark brown. Palpomeres cylindrical with last segment long-oval; third segment shortest, remaining segments almost equal in length. Setae on palpus dark brown.

**Figure 24:**
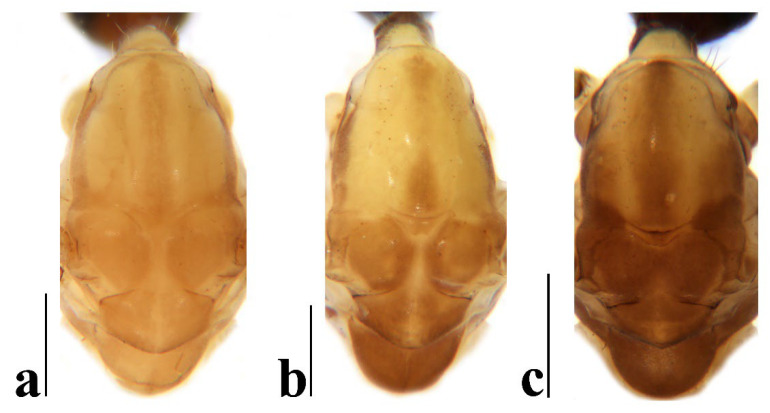
*Discobola taivanella* (Alexander, 1930). (**a**–**c**) Variations of thorax, dorsal view. Scale bars: 0.5 mm (**a**–**c**).

**Figure 25:**
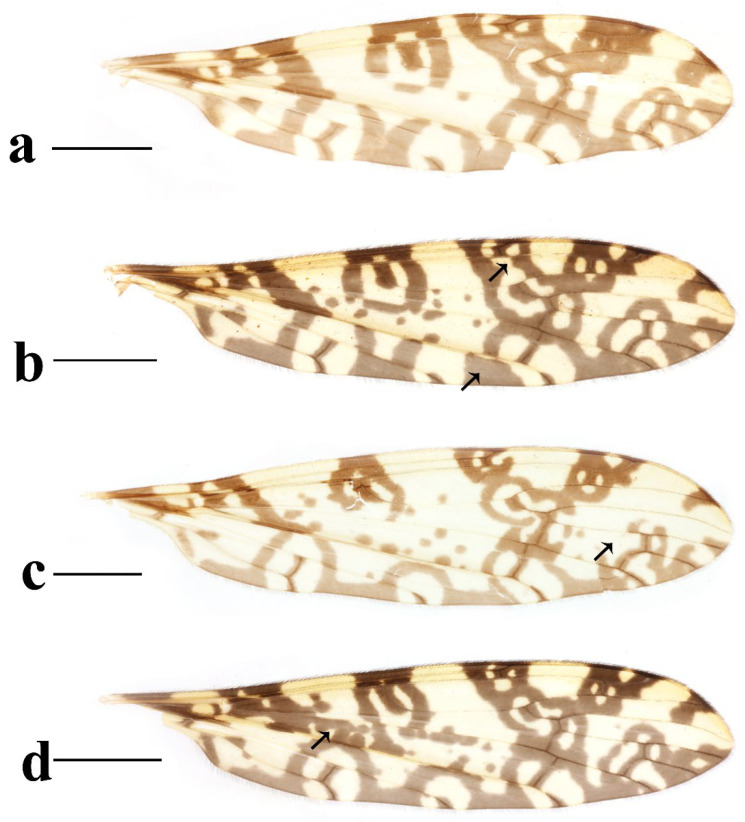
*Discobola taivanella* (Alexander, 1930). (**a**–**d**) Variations of wing. The black arrows indicate the variable wing spots. Scale bars: 1.5 mm (**a**–**d**).

Thorax (Figure 23a and Figure 24). Pronotum yellow. Prescutum and presutural scutum yellow with lateral margins light brown, middle with a yellowish brown stripe (Figure 24a); prescutum and presutural scutum in some specimens with more darker lateral margins and broken middle light brown stripe (Figure 24b) or with three broad brown stripes (Figure 24c). Postsutural scutum yellow, each lobe with one yellowish brown spot (Figure 24a); postsutural scutum in some specimens yellow, each lobe with one light brown spot (Figure 24b); postsutural scutum in some specimens uniformly brown with posterior margin yellow (Figure 24c). Scutellum yellowish brown (Figure 24a); scutellum in some specimens light brown with middle area yellow (Figure 24b) or brown with posterior area yellow (Figure 24c). Mediotergite yellowish brown (Figure 24a) or brown (Figure 24b,c). Pleuron (Figure 23a) yellow, with two longitudinal narrow brown stripes. Setae on thorax brown. Coxae and trochanters yellow; femora yellow basally and gradually darkened to brown, tip with two clear broad yellow rings, enclosing a broad brown ring; tibiae yellow with tips darker; tarsi yellow. Setae on legs yellowish brown. Wing (Figure 1f and Figure 25) pale yellow, with usual brown ocellate pattern as follows: base of wing, base of Rs, sc-r, R_2_, fork of R_4+5_, r-m, m-m, m-cu, tip of CuP, tip of A_1_ (Figure 25a); ocellate pattern in sc-r and m-m sometimes reduced (Figure 25b,c); ocellate pattern in tip of CuP sometimes enlarged (Figure 25b). Wing with one large brown spot at tip of R_4_. Wing also with some spots covered in cell r and m, sometimes spots fused (Figure 25d). Veins brown, darker in pattern areas. Venation: Sc before fork of Rs; tip of R_1_ slightly curved; basal section of M_3_ about 1.5 to 2 times length of m-m; r-m about 1/3–1/2 length of inner edge of dm; m-cu slightly before or beyond the fork of M. Stem of halter dark brown, base and tip yellow; knob pale yellow (Figure 23a,c).

Abdomen (Figure 23a). Segment 1 yellow with caudal margin brown. Tergites 2–7 yellow with medial area and caudal margin brown, tergite 8 yellow with caudal margin brown. Sternites 2–7 yellow with lateral and caudal margin brown, sternite 8 brown. Setae on abdomen brown.

**Figure 26:**
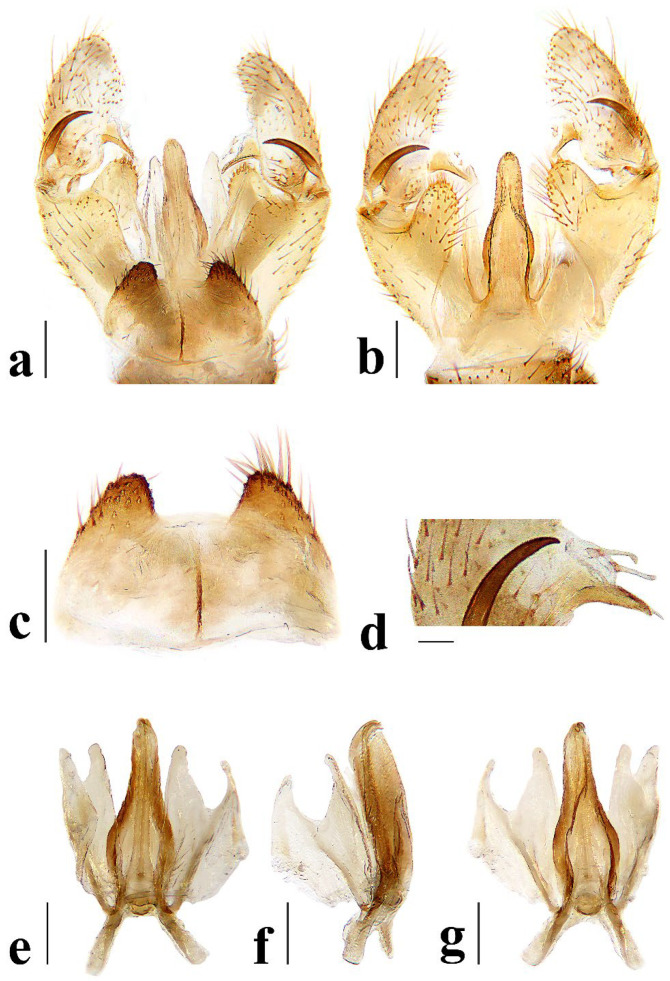
*Discobola taivanella* (Alexander, 1930). (**a**) Male hypopygium, dorsal view; (**b**) male hypopygium, ventral view; (**c**) tergite 9, dorsal view; (**d**) rostral prolongation, dorsal view; (**e**) complex of aedeagus, dorsal view; (**f**) complex of aedeagus, lateral view; (**g**) complex of aedeagus, ventral view. Scale bars: 0.2 mm (**a**–**c**,**e**–**g**); 0.05 mm (**d**).

Hypopygium (Figure 26) yellow. Tergite 9 (Figure 26a,c) trapeziform, posterior margin with board U-shaped notch; each lobe with dense long brown setae. Gonocoxite (Figure 26a,b) cylindrical, middle with an elongated ventromesal lobe; gonocoxite with short brown setae, ventromesal lobe with dense long brown setae. Outer gonostylus (Figure 26a,b) narrow, arched with tip acute and sclerotized. Inner gonostylus (Figure 26a,b) about twice length of outer gonostylus, long-oval. Rostral prolongation (Figure 26a,b,d) short and slender, near base bearing tubercles armed with two separated spines. Paramere (Figure 26e–g) finger-shaped, wide at base, narrowed and curved outward apically, curved outward. Aedeagus (Figure 26a,b,e–g) cylindrical, widened at base, slightly narrowed at middle.

**Figure 27:**
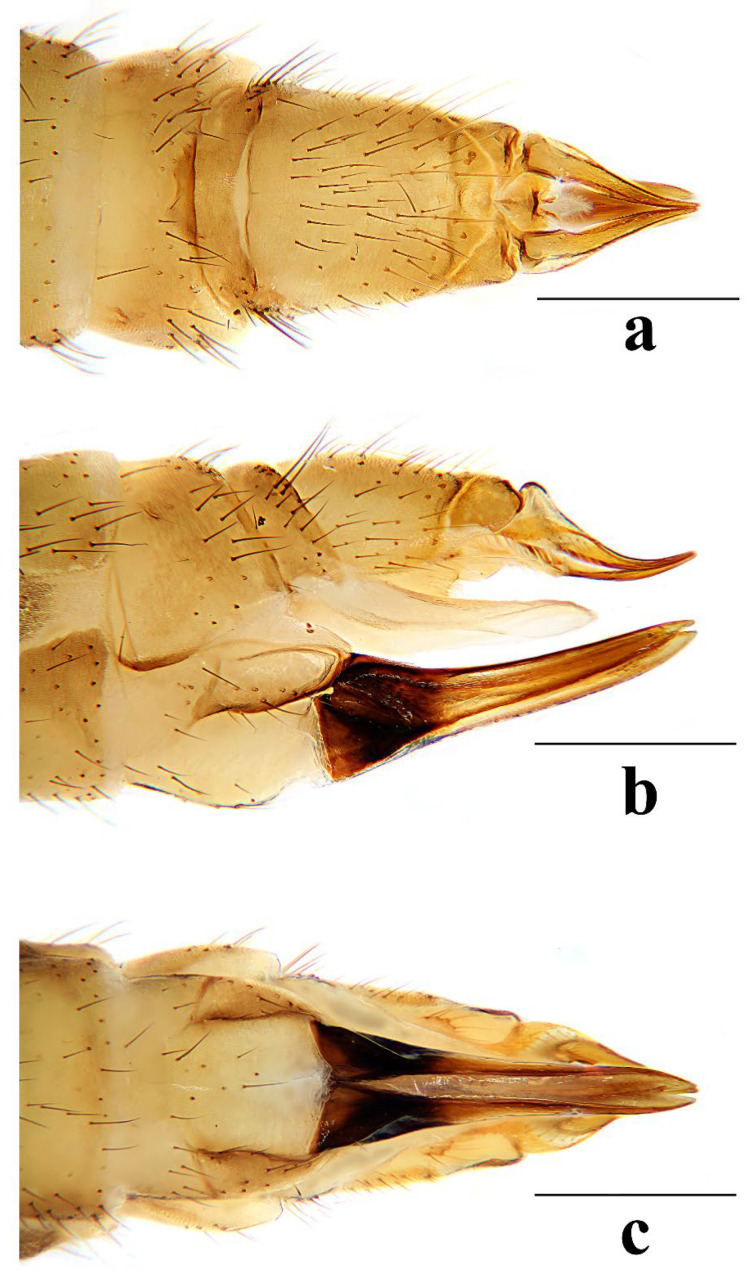
*Discobola taivanella* (Alexander, 1930). (**a**) Female ovipositor, dorsal view; (**b**) female ovipositor, lateral view; (**c**) female ovipositor, ventral view. Scale bars: 0.5 mm (**a**–**c**).

Female. Body length 7.1–10.7 mm, wing length 7.2–11.0 mm. Generally similar to male by body coloration. Ovipositor (Figure 27) with tergite 9 yellow. Tergite 10 yellowish brown, slightly darker in caudal margin. Cercus short, yellowish brown with tip darker (Figure 27a,b). Hypogynial valve brown, with 1/3 brownish black at base (Figure 27b,c).

Specimens examined. Holotype, male (USNM), China: Taiwan, Hassensan (1981.2 m–2286 m), 31 August 1929, S. Issiki. Other materials: 1 male 2 females, China: Beijing Municipality, Mentougou District, Qingshui Town, Xiaolongmen Science Experimental Zone (1176 m), 20 September 2017, Hu Li (Malaise trap). 1 male, China: Beijing Municipality, Mentougou District, Qingshui Town, Xiaolongmen Science Experimental Zone (39°57′46″ N, 115°25′51″ E, 1192 m), 20 September 2017, Hu Li (Malaise trap). 1 male, China: Beijing Municipality, Mentougou District, Xiaolongmen National Forest Park, 16 September 2012, Yuyu Wang (Malaise trap). 1 male, China: Beijing Municipality, Mentougou District, Qingshui Town, Lingshan Natural Scenic Area, Lingshan Meadow (1552m), 20 September 2019, Hu Li (Malaise trap). 1 male, China: Beijing Municipality, Mentougou District, Qingshui Town, Xiaolongmen Science Experimental Zone, 20 September 2017. 2 males 1 female, China: Fujian Province, Mount Wuyi, Huanggangshan Primeval Forest Nature Park (1812 m), 27 August 2023, Zehui Kang. 1 male 2 females, China: Fujian Province, Mount Wuyi, Huanggangshan Primeval Forest Nature Park (1872 m), 27 August 2023, Zehui Kang. 2 males, China: Gansu Province, Diebu County, Duoer Forest Farm, Houxizanggou (2733 m), 18 August 2016, Liang Wang. 1 male, China: Guangxi Autonomous Region, Longsheng county, Huaping National Nature Reserve, Anjiangping, 6 June 2021 (Malaise Trap). 1 male, China: Guangxi Autonomous Region, Longsheng county, Huaping National Nature Reserve, Anjiangping, 20 June 2021 (Malaise Trap). 1 female, China: Guangxi Autonomous Region, Longsheng county, Huaping National Nature Reserve, Anjiangping, 4 July 2021 (Malaise Trap). 1 female, China: Guangxi Autonomous Region, Longsheng county, Huaping National Nature Reserve, Anjiangping, 2021 (Malaise Trap). 1 male 1 female, China: Guizhou Province, Mount Fanjing, Jinding (2100m), 31 May 2002, Ding Yang. 1 male 1 female, China: Guizhou Province, Mount Fanjing (1800m), 3 June 2002, Ding Yang. 1 female, China: Guizhou Province, Leishan County, Leigongshan Protection Station (1528m), 18 July 2014, Yue Lu (light trap). 1 male, China: Henan Province, Yiyang County, Mount Huaguo, 5 August 2015, Shan Huo. 1 male, China: Hunan Province, Sangzhi County, Mount Doupeng (1680 m), 6 August 2015, Yuqiang Xi (Malaise trap). 3 females, China: Hunan Province, Sangzhi County, Mount Doupeng (1680 m), 4 August 2015, Wenmin Xiao. 1 male, China: Hunan Province, Sangzhi County, Mount Tianping (1500 m), 18 June 2014, Xiao Zhang. 1 male, China: Hunan Province, Junshan District, Dongting Lake, Caisang Lake, 19 October 2007, Kuiyan Zhang. 2 males 1 female, China: Shaanxi Province, Feng County, near Xihe Temple (1532.5 m), 24 June 2024, Hanhuiying Lv (light trap). 1 male, China: Sichuan Province, Emeishan City, Mount Emei, Linggongli, 15 July 2010, Tao Li. 1 male, China: Sichuan Province, Pingwu County, Wanglang National Nature Reserve (2500 m), 31 July 2017, Yuqiang Xi. 1 male, China: Sichuan Province, Wenchuan County, Wolong National Nature Reserve, Dengshenggou (2719 m), 7 August 2013, Xiaoyan Liu. 1 male, China: Shanxi Province, Qinshui County, Lishan National Nature Reserve, Liwangping, 30 July 2013, Shuai Su. 1 female, China: Xizang Autonomous Region, Linzhi City, 12 to 22 September 2012 (Malaise trap). 1 female, China: Xizang Autonomous Region, Linzhi City, 2 to 27 September 2012 (Malaise trap). 1 male, China: Xizang Autonomous Region, Linzhi City, 22 September 2012 to 1 October 2012 (Malaise trap). 2 females, China: Yunnan Province, Nanjian County, Mount Wuliang (2221 m), 16 July 2016, Qicheng Yang (light trap). 1 female, China: Yunnan Province, Nanjian County, Mount Wuliang (2221 m), 16 July 2016, Qicheng Yang. 1 female, China: Yunnan Province, Nanjian County, Mount Wuliang (2281 m), 17 July 2016, Qicheng Yang. 1 female, China: Zhejiang Province, Longquan City, Mount Fengyang, Datianping Warehouse (1290 m), 9 November 2007, Shenglong Liu. 2 females, China: Zhejiang Province, Longquan City, Mount Fengyang, Longquan falls (1520 m), 8 October 2008, Shenglong Liu. 1 male, China: Zhejiang Province, Hangzhou City, Mount Tianmu, 12 August 2008, Gang Yao (Malaise trap). 1 female, China: Zhejiang Province, Qingyuan County, Baishanzu (1523 m), 25 July 2021, Qicheng Yang.

Distribution. China (Beijing, Fujian, Gansu, Guangxi, Guizhou, Henan, Hunan, Shaanxi, Shanxi, Sichuan, Taiwan, Xizang, Yunnan, Zhejiang).

#### 3.1.7. Key to Chinese *Discobola* Species

1 Wing without usual ocellate pattern, instead with large dark brown areas and spots (Figure 2c) ........................................................................................*Discobola acurostris* (Alexander, 1943)

- Wing with usual ocellate pattern (Figure 5, Figure 10, Figure 15, Figure 20 and Figure 25) ............................................................................................................................................................... 2

2 Prescutum and presutural scutum without stripes (Figure 14); wing with ocellate pattern bicolored (Figure 15); inner gonostylus of male hypopygium with two long and stout setae at tip (Figure 16a,b,d) ............................................................................................................................................................................................................................ *Discobola margarita* Alexander, 1924

- Prescutum and presutural scutum with at least one stripe (Figure 4, Figure 9, Figure 19 and Figure 24); wing with ocellate pattern unicolor (Figure 5, Figure 10, Figure 20 and Figure 25); inner gonostylus of male hypopygium without long and stout seta at tip (Figure 6, Figure 11, Figure 21 and Figure 26) ........................................................................................................3

3 Wing without small spots in cell m (Figure 5); pleuron with three stripes (Figure 3a) ............................................................................................................ *Discobola annulata* (Linnaeus, 1758)

- Wing with more or less small spots in cell m (Figure 10, Figure 20 and Figure 25); pleuron with less than three stripes (Figure 8a, Figure 18a and Figure 23a) ..............................................4

4 Wing with broken ocellate pattern (Figure 20); inner gonostylus as long as outer gonostylus (Figure 21a); rostral prolongation bearing a very small and peg-shaped spine near base (Figure 21d) .......................................................................................................................................................................................................................... *Discobola parvispinula* (Alexander, 1947)

- Wing with usual ocellate pattern (Figure 10 and Figure 25); inner gonostylus at least 1.5 times length of outer gonostylus (Figure 11a and Figure 26a); rostral prolongation bearing tubercles armed with two separated spines near base (Figure 11d and Figure 26d) ............................................................................................................................................................................. 5

5 Pleuron with two stripes (Figure 23a); wing with small spots only in cell r and m (Figure 25); knob of halter pale yellow (Figure 23c); inner gonostylus about twice length of outer gonostylus (Figure 26a,b) ............................................................................................................................................................................................................... *Discobola taivanella* (Alexander, 1930)

- Pleuron with one stripe (Figure 8a); wing with small spots in cell r_1_, cell r, m, dm, cua, and a_1_ (Figure 10); knob of halter pale white, base brown (Figure 8c); inner gonostylus about 1.5 length of outer gonostylus (Figure 11a,b) ....................................................................................................................................................................................... *Discobola armorica* (Alexander, 1942)

### 3.2. Distribution of Discobola in China

**Figure 28:**
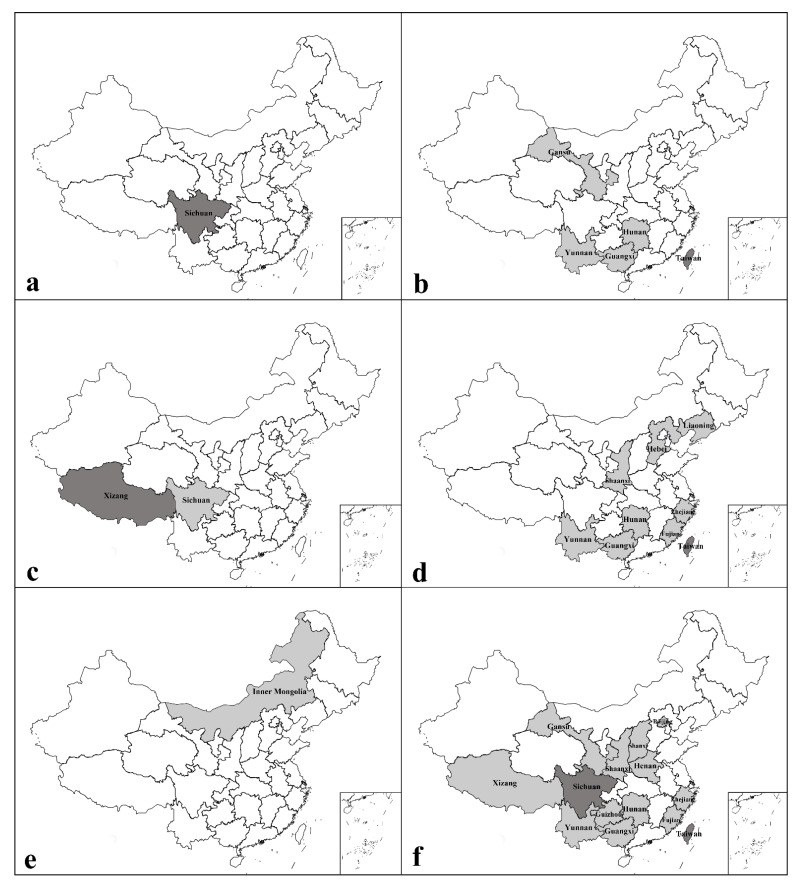
Distribution maps of *Discobola* species in China (dark gray areas: previously known distribution records; light gray areas: new distribution records in this study). (**a**) *Discobola acurostris* (Alexander, 1943); (**b**) *Discobola annulata* (Linnaeus, 1758); (**c**) *Discobola armorica* (Alexander, 1942); (**d**) *Discobola margarita* Alexander, 1924; (**e**) *Discobola parvispinula* (Alexander, 1947); (**f**) *Discobola taivanella* (Alexander, 1930).

According to Oosterbroek (2025) [1], the genus *Discobola* was previously known from only three provincial-level regions in China, with five known species. In the present study, an additional species of *Discobola* is recorded from China for the first time, and the distributional data for this genus are substantially expanded (Figure 28). Based on a synthesis of published literature [1] and new data from this study, six *Discobola* species are now confirmed to occur in China, distributed across 17 provincial-level regions. Among the six known Chinese species, *D. taivanella* (Alexander, 1930), *D. margarita* Alexander, 1924 and *D. annulata* (Linnaeus, 1758) display broad geographic distributions, having been recorded in 14, 9 and 5 provincial-level regions, respectively (Figure 28b,d,f). In contrast, *D. armorica* (Alexander, 1942) has a more restricted range, confirmed only from Sichuan and Xizang (Figure 28c). The remaining two species, *D. acurostris* (Alexander, 1943) and *D. parvispinula* (Alexander, 1947), are each known only from a single provincial-level region—Sichuan and Inner Mongolia, respectively (Figure 28a,e).

**Figure 29:**
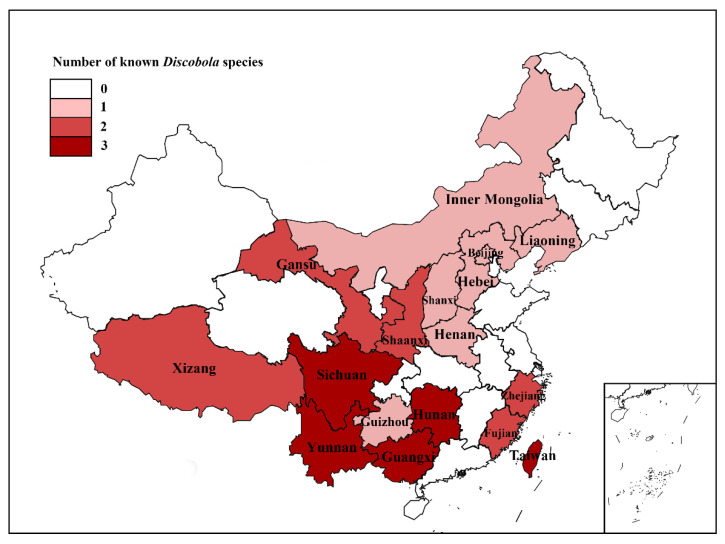
Distribution map of the genus *Discobola* Osten Sacken, 1865 in China with species richness.

Analysis of species richness across China reveals that five provincial-level regions—Guangxi, Hunan, Sichuan, Taiwan and Yunnan—harbor the highest species richness, each with three recorded *Discobola* species (Figure 29). Notably, four of these regions (i.e., Guangxi, Hunan, Taiwan and Yunnan) share the same three species: *D. annulata* (Linnaeus, 1758), *D. margarita* Alexander, 1924 and *D. taivanella* (Alexander, 1930), all of which are widely distributed throughout the country (Figure 28b,d,f). In contrast, Sichuan exhibits a distinct species composition, comprising *D. annulata* (Linnaeus, 1758), the more narrowly distributed *D. armorica* (Alexander, 1942), and the regionally endemic *D. acurostris* (Alexander, 1943) (Figure 28a–c). Several other provinces, including Fujian, Gansu, Shaanxi, Xizang and Zhejiang, are each home to two species of *Discobola*. The remaining provincial-level regions either have a single recorded species or no confirmed distribution records to date (Figure 29).

Overall, the current distribution pattern indicates higher species richness in southern China and the Qinghai-Tibet region, with a gradual decline in diversity toward the northern and northwestern provinces. This biogeographic trend may be attributed to the complex and heterogeneous topography of southern China and the Qinghai-Tibet region, which includes prominent landforms such as the Qinghai-Tibet Plateau, the Sichuan Basin, the Hengduan Mountains and the Shiwandashan Mountains. These ecologically diverse landscapes likely offer a variety of microhabitats and environmental conditions that promote allopatric speciation and endemism in *Discobola* crane flies. Southern China is characterized by subtropical to tropical humid climates and supports diverse vegetation zones, including evergreen broadleaf forests and montane seasonal rainforests. These forest ecosystems provide favorable conditions for *Discobola* crane flies, including high humidity, shaded microhabitats and abundant organic matter essential for larval development and adult survival. Similarly, although the Qinghai-Tibet Plateau is dominated by a high-altitude mountain climate, its pronounced altitudinal zonation supports a mosaic of vegetation types ranging from temperate mixed forests at lower elevations to alpine shrubs and meadows at higher elevations, offering ecological niches for *Discobola* crane flies. In contrast, northern and northwestern China are predominantly characterized by temperate arid to semi-arid climates, with steppe, shrubland, and desert vegetation types, which generally lack the stable, moist environments suitable for sustaining *Discobola* populations. Additionally, many of these regions are situated within or adjacent to one of the world’s recognized biodiversity hotspots [66], further underscoring their ecological importance. Although future studies and additional field surveys are expected to reveal new distribution records and potentially undiscovered species, the present findings significantly enhance our understanding of the diversity and biogeography of *Discobola* in China, and provide a valuable foundation for future research in taxonomy, ecology and evolutionary biology of this genus.

### 3.3. Molecular Phylogeny and DNA Barcoding of Discobola

**Figure 30:**
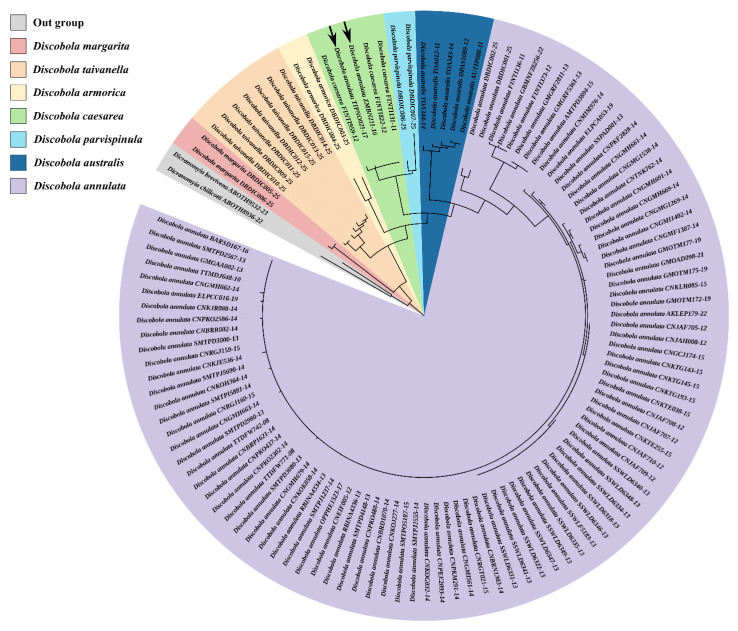
Maximum likelihood tree of all known *COI* sequences in the genus *Discobola* Osten Sacken, 1865. The black arrows refer to two nested sequences of *D. annulata* (Linnaeus, 1758) in the *D. caesarea* (Osten Sacken, 1854) clade.

In the present study, we newly generated 15 mt *COI* sequences (average length ≈ 540 bp) from five *Discobola* species collected in China, which represent the first DNA barcode reference library established specifically for Chinese *Discobola* species. The ML phylogenetic tree, constructed using 118 mt *COI* sequences obtained from both newly generated and publicly available data, is shown in Figure S1 and Figure 30. Among the 118 sequences, 116 correspond to seven species of *Discobola* and are designated as the ingroup, while the remaining two sequences, representing two *Dicranomyia* Stephens, 1829 (Diptera: Limoniidae) crane flies, are assigned as outgroup taxa. The ingroup is resolved into seven distinct clades, among which the *D. caesarea* (Osten Sacken, 1854) clade contains two nested sequences of *D. annulata* (Linnaeus, 1758), while each of the remaining six clades comprises sequences belonging exclusively to a single species.

Genetic distances of the 116 *Discobola* mt *COI* sequences are presented in Table S2 and Table 2. The genetic distances between *D. caesarea* (Osten Sacken, 1854) sequences and the two nested sequences of *D. annulata* (Linnaeus, 1758) range from 0 to 0.6%, indicating that they may represent the same species. Due to the potential misidentification of the two *D. annulata* (Linnaeus, 1758) sequences, they were excluded from subsequent analysis of intra- and interspecific divergence. Genetic distance analysis based on the remaining 114 *Discobola* sequences indicates that intraspecific genetic distances within *Discobola* are generally below 7.4%, with the highest value observed in *D. annulata* (Linnaeus, 1758). Notably, the interspecific distance between *D. caesarea* (Osten Sacken, 1854) and *D. parvispinula* (Alexander, 1947) ranges from 2.4% to 5.5%, which is lower than the maximum intraspecific divergence observed within the genus. Taken together with the strongly supported (bootstrap support = 99%) sister-group relationship between the two species in the phylogenetic tree, the low interspecific divergence suggests the possibility of either misidentification or that the two taxa may be conspecific, representing a case of taxonomic synonymy. When excluding comparisons between *D. caesarea* (Osten Sacken, 1854) and *D. parvispinula* (Alexander, 1947), the minimum interspecific distance within the genus is 7.6%, observed between *D. annulata* (Linnaeus, 1758) and *D. australis* (Skuse, 1890), as well as between *D. annulata* (Linnaeus, 1758) and *D. parvispinula* (Alexander, 1947). In contrast, the maximum interspecific distance reaches 17.7%, occurring between *D. annulata* (Linnaeus, 1758) and *D. margarita* Alexander, 1924.

For comparison, a previous study reported that within the related genus *Limonia* Meigen, 1803 (Limoniidae), intraspecific genetic distances are no greater than 2.2%, whereas interspecific distances range from 7.9% to 17.2% [56]. The interspecific distances observed in *Discobola* (7.6–17.7%) are broadly consistent with those in *Limonia*, however, the maximum intraspecific distance in *Discobola* (7.4%) is markedly higher. This discrepancy may be attributed to intergeneric variation in genetic divergence patterns within Limoniidae. In *Discobola*, species with broad geographic distributions often exhibit high levels of genetic divergence among geographically isolated populations. These results highlight the need for future integrative taxonomic studies that combine molecular, morphological and geographic data to improve species delimitation within *Discobola*. Such approaches will be essential for resolving taxonomic uncertainties and understanding patterns of genetic diversity in this genus.

## 4. Conclusions

In the present study, six Chinese species of the genus *Discobola* are redescribed and illustrated, including one species newly recorded from China. Furthermore, the distributional data of the genus in China are substantially updated, with its known range expanding from three to seventeen provincial-level regions across the country. This expanded distribution reveals greater species richness in southern China and the Qinghai–Tibet region, with a gradual decrease in species richness toward northern and northwestern China. The molecular data generated in this study, based on mt *COI* barcodes, are congruent with morphological identifications. This research establishes the first DNA barcode reference library for Chinese *Discobola*, thereby contributing to the continuously expanding global DNA barcode library of crane flies. DNA barcoding analysis reveals that intraspecific distances within this genus are generally less than 7.4%, while interspecific distances range from 7.6% to 17.7%. Although no overlap is observed between intra- and interspecific divergences, the narrow gap between them suggests potential limitations in using mt *COI* barcodes alone for precise species delimitation within the genus. Therefore, further integrative taxonomic studies incorporating in-depth morphological examinations, comparative analyses of geographically diverse populations, and a broader spectrum of molecular markers will be essential to improve the resolution of species boundaries and to understand evolutionary relationships within *Discobola*.

## Figures and Tables

**Table 1:** Information of *Discobola* specimens sequenced in this study.

Sample ID	Species	Sex	Locality	Accession Number
DIAN01	*D. annulata*	male	CHINA: Yunnan Province, Gongshan County	DBDIC001-25
DIAN02	*D. annulata*	male	CHINA: Yunnan Province, Nanjian County, Mount Wuliang (2221 m)	DBDIC002-25
DIAR01	*D. armorica*	female	CHINA: Sichuan Province, Pingwu County, Wanglang National Nature Reserve, Great Lawn (2930 m)	DBDIC003-25
DIAR02	*D. armorica*	female	CHINA: Xizang Autonomous Region, Bomi County, Bagai Township (3045 m)	DBDIC004-25
DIMA01	*D. margarita*	female	CHINA: Guangxi Autonomous Region, Longsheng County, Huaping National Nature Reserve, Anjiangping	DBDIC005-25
DIMA02	*D. margarita*	male	CHINA: Shaanxi Province, Feng County, near Tongtianhe National Forest Park (1551.5 m)	DBDIC006-25
DIPA01	*D. parvispinula*	female	CHINA: Inner Mongolia Autonomous Region, Genhe County, Genhe Aoluguya Airport (685.2 m).	DBDIC007-25
DIPA02	*D. parvispinula*	male	CHINA: Inner Mongolia Autonomous Region, Harqin Banner, Wangyedian National Forest Park (1392–1620 m)	DBDIC008-25
DITA01	*D. taivanella*	male	CHINA: Shaanxi Province, Feng County, near Xihe Temple (1532.5 m)	DBDIC009-25
DITA02	*D. taivanella*	male	CHINA: Hunan Province, Sangzhi County, Mount Doupeng (1680 m)	DBDIC010-25
DITA03	*D. taivanella*	male	CHINA: Beijing Municipality, Mentougou District, Qingshui Town, Xiaolongmen Science Experimental Zone (39°57′46″ N, 115°25′51″ E, 1192 m)	DBDIC011-25
DITA04	*D. taivanella*	male	CHINA: Fujian Province, Mount Wuyi, Huanggangshan Primeval Forest Nature Park (1872 m)	DBDIC012-25
DITA05	*D. taivanella*	female	CHINA: Guangxi Autonomous Region, Longsheng County, Huaping National Nature Reserve, Anjiangping	DBDIC013-25
DITA06	*D. taivanella*	female	CHINA: Guizhou Province, Leishan County, Leigongshan Protection Station (1528 m)	DBDIC014-25
DITA07	*D. taivanella*	female	CHINA: Zhejiang Province, Longquan City, Mount Fengyang, Datianping Warehouse (1290 m)	DBDIC015-25

**Table 2:** Genetic distances between *Discobola* Osten Sacken, 1865 mt *COI* sequences.

Species (Number of Sequences)	*D. annulata* (93)	*D. armorica* (2)	*D. australis* (5)	*D. caesarea* (3)	*D. margarita* (2)	*D. parvispinula* (2)	*D. taivanella* (7)
***D. annulata* (93)**	0–7.4% *						
***D. armorica* (2)**	9.3–11.3% ^#^	4.4% *					
***D. australis* (5)**	7.6–11.8% ^#^	10.0–12.0% ^#^	0.2–1.7% *				
***D. caesarea* (3)**	8.0–11.7% ^#^	10.9–11.7% ^#^	10.4–10.9% ^#^	0–0.4% *			
***D. margarita* (2)**	15.2–17.7% ^#^	14.2–16.0% ^#^	14.4–15.8% ^#^	12.6–13.7% ^#^	0.4% *		
***D. parvispinula* (2)**	7.6–12.7% ^#^	9.9–14.3% ^#^	10.9–12.2% ^#^	2.4–5.5%	13.9–14.7% ^#^	4.6% *	
***D. taivanella* (7)**	7.8–10.8% ^#^	8.6–10.3% ^#^	9.5–10.7% ^#^	8.3–9.2% ^#^	11.6–13.4% ^#^	8.7–10.2% ^#^	0.2–3.3% *

* Intraspecific distances. ^#^ Interspecific distances.

## Data Availability

The mitochondrial *COI* sequences obtained in this study are publicly available in the BOLD database [59] (http://www.boldsystems.org/) under accession numbers DBDIC001-25 to DBDIC015-25, where only the numerical portion preceding the hyphen increases sequentially.

## Supplementary Materials

The following supporting information can be downloaded at: https://www.mdpi.com/article/10.3390/insects16080845/s1, Figure S1: Maximum Likelihood tree of all known *COI* sequences in the genus *Discobola* Osten Sacken, 1865. The number at each node is the posterior probability (PP); Table S1. Information of species used in phylogenetic analysis with BOLD accession numbers; Table S2. Details of genetic distances between *Discobola* Osten Sacken, 1865 *COI* sequences.
