# Supplementary figures and images for: Discobola Osten Sacken, 1865 (Diptera, Limoniidae) in China: Taxonomic Review, Updated Distribution, and DNA Barcoding
Source: Insects. 2025 Aug 15;16(8):845. doi: 10.3390/insects16080845 (PMC12386808; doi:10.3390/insects16080845)

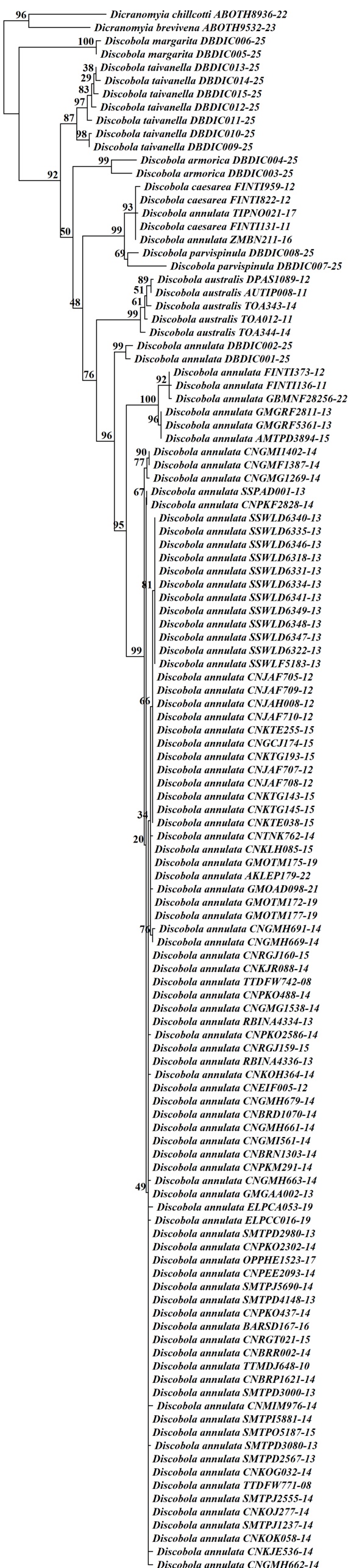

0.050

Supplement: Supplementary file 1 [file insects-16-00845-s001.zip › Figure S1.pdf]
